# Metal tolerance of Río Tinto fungi

**DOI:** 10.3389/ffunb.2024.1446674

**Published:** 2024-10-16

**Authors:** Monike Oggerin, Catalina del Moral, Nuria Rodriguez, Nuria Fernandez-Gonzalez, José Manuel Martínez, Iván Lorca, Ricardo Amils

**Affiliations:** ^1^ Centro de Biología Molecular Severo Ochoa (CBMSO, CSIC-UAM), Universidad Autónoma de Madrid, Madrid, Spain; ^2^ Centro de Astrobiología (CAB, INTA-CSIC), Madrid, Spain; ^3^ Centro Nacional de Biotecnología (CSIC), Universidad Autónoma de Madrid, Madrid, Spain

**Keywords:** acidic fungi, Río Tinto, heavy metals, Eurotiomycetes, Dothideomycetes, Sordariomycetes, *Penicillium*, *Acidiella*

## Abstract

Southwest Spain’s Río Tinto is a stressful acidic microbial habitat with a noticeably high concentration of toxic heavy metals. Nevertheless, it has an unexpected degree of eukaryotic diversity in its basin, with a high diversity of fungal saprotrophs. Although some studies on the eukaryotic diversity in Rio Tinto have been published, none of them used molecular methodologies to describe the fungal diversity and taxonomic affiliations that emerge along the river in different seasons. The aim of the present study was to isolate and describe the seasonal diversity of the fungal community in the Río Tinto basin and its correlation with the physicochemical parameters existing along the river’s course. The taxonomic affiliation of 359 fungal isolates, based on the complete internal transcribed spacer DNA sequences, revealed a high degree of diversity, identifying species belonging primarily to the phylum Ascomycota, but representatives of the Basidiomycota and Mucoromycota phyla were also present. In total, 40 representative isolates along the river were evaluated for their tolerance to toxic heavy metals. Some of the isolates were able to grow in the presence of 1000 mM of Cu^2+^, 750 mM of As^5+^ and Cd^2+^, and 100 mM of Co^2+^, Ni^2+^, and Pb^2+^.

## Introduction

**Figure 1:**
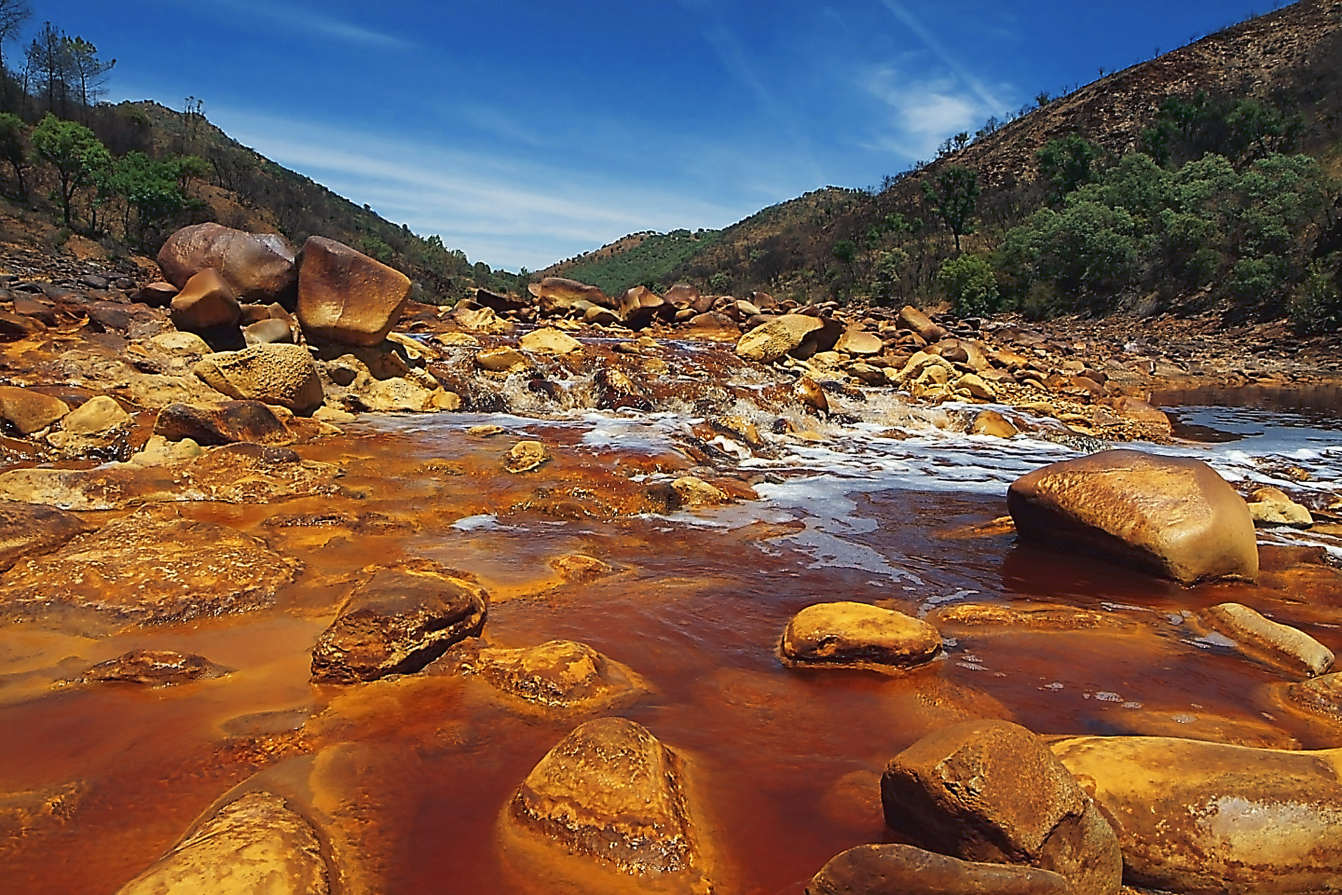
Río Tinto at Berrocal.

Río Tinto (Southwestern Iberian Peninsula) (**Figure 1**) is a natural rock drainage system enhanced by the subsurface bio-oxidation of the volcanogenic massive sulfide ores by chemolithotrophic prokaryotes (Amils et al., 2023; Gómez-Ortiz et al., 2014) within the Iberian Pyrite Belt (IPB). Río Tinto drains from the giant volcanogenic massive sulfides of the homonymous district. There, up to 500 Mt of the massive sulfides, made up predominantly of pyrite with minor amounts of chalcopyrite, sphalerite, and galena, as well as nearly 2000 Mt of underlying pyrite-bearing stockwork, make up the largest known sulfide anomaly on the Earth’s surface (Tornos, 2006). As a consequence, the generation of high concentrations of ferric iron, a strong oxidant, facilitates the dissolution of heavy metals (Cu, Zn, As, and Mn) during the oxidation of sulfides which, due to its buffering potential (González-Toril et al., 2003) maintain the constant pH detected along the 92-km river. What makes Río Tinto remarkable to a microbiologist is the unexpected degree of eukaryotic diversity found in its waters (Amaral Zettler et al., 2002; López-Archilla et al., 2001; Aguilera et al., 2006), with fungi the most diverse among the decomposers (López-Archilla et al., 2001, 2004). Most of the river biomass is located on the riverbed, forming dense and compact biofilms (Aguilera et al., 2007a).

In extremely acidic environments with a high content of toxic metals, such as the Tinto ecosystem, fungal survival and growth are possible due to a range of strategies developed for metal resistance and tolerance (Gadd, 2008, 2021; Lowenstam, 1981; Miersch et al., 2001; Sharples et al., 2001). These include synthesis of chelating agents, complexation and crystallization, decreased transport or impermeability by cell wall thickening, intracellular compartmentalization and precipitation, biosorption to cell walls, or biomineralization, among others (e.g. Brown and Hall, 1990; Gadd, 1990, 2008). The best-characterized mechanisms that Río Tinto fungi have developed to survive in the presence of high concentrations of toxic heavy metals are unspecific sequester in the cellular cell walls, specific intracellular sequester (Durán et al., 1999), active transport (López-Archilla et al., 2004; Oggerin et al., 2015), and biomineralization processes (Oggerin et al., 2013, 2016). Most of these mechanisms are related to biologically induced mineralization (Lowenstam, 1981) because the negatively charged cell walls are able to bind metallic cations through non-specific electrostatic interactions, acting as adsorption sites for ions that facilitate nucleation and mineral growth, which contribute to local supersaturation and decrease the free energy barriers (Bazylinski, 2001; Beveridge, 1989; Konhauser, 1998; Lowenstam, 1981). Thus, in addition to growth conditions, the resultant minerals depend on the nature of the cell surface, the cellular microenviroment, and the presence of certain reactive anions in the cell walls (Gadd, 2021). Because electrostatic interactions are metabolically independent, the cells do not even have to be viable. Dead cells retain the ability to bind metallic ions (Beveridge and Murray, 1976; Urrutia Mera et al., 1992). These capabilities could lead to useful methodologies for separating and recovering valuable heavy metals from contaminated industrial wastewater (Liang and Gadd, 2017; Ferrier et al., 2021; Gadd, 2021).

Although different studies describing the outstanding eukaryotic diversity identified along the Tinto basin have been published, none of them have provided a description of the fungal diversity or their taxonomic affiliation along the entire length of the river during all four seasons (Amaral Zettler et al., 2002; López-Archilla et al., 2001, 2004). The aim of the present study was to isolate and describe the diversity of the fungal community in the extremely acidic environment of the Tinto basin during different seasons, along with the correlation of the physicochemical parameters existing along the river´s course. Molecular diversity studies were performed using the complete ITS region (ITS1-5.8S-ITS2). Taxonomic affiliation showed a high degree of diversity, and selected isolates were evaluated for their tolerance to toxic heavy metals in order to assess their biotechnological potential for the bioremediation of heavy metal-contaminated wastewater.

## Materials and methods

### Sampling sites and collection

**Figure 2:**
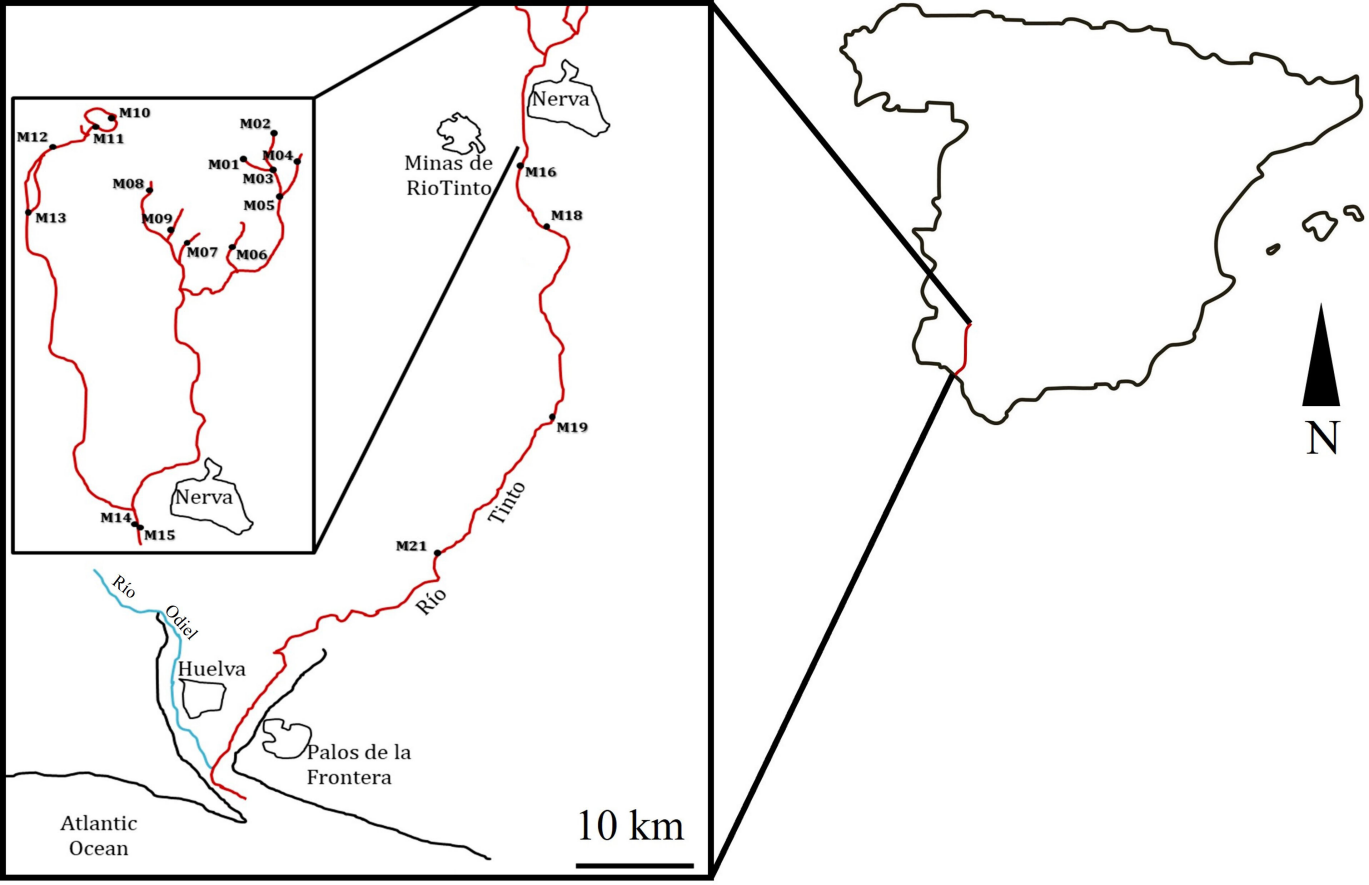
Location of the study area, showing the different sampling sites along the Río Tinto. The scale corresponds to 10 km.

In total, 19 sites along the river were selected for *in situ* measurements and sampling (**Figure 2**). Water and sediment samples were collected in March (24-27), June (9-12), and December (15-18) of 2009; March (15-18), June (7-10), and December (6-9) of 2010; and March (21-24) of 2011 at the selected sampling stations (**Table 1**). Climatic data can be found in the following links: https://www.miteco.gob.es/es/cartografia-y-sig/ide/ and https://www.aemet.es/es/serviciosclimaticos/datosclimatologicos. The physicochemical parameters (temperature, water conductivity, redox potential, pH, and dissolved oxygen) were measured *in situ* using YSI 556 MPS multi-parametric probes. The probes were calibrated on each sampling day. For pH, H154710-11 and H15002 (HANNA) kits were used. Conductivity and salinity were calibrated with the standard solutions H17030M and H17034M. Elemental analysis was conducted at the Scientific Service of the Universidad Autónoma de Madrid.

**Table 1:** Coordinates and environmental data averages of the 19 sampling sites over 3 years.

Sampling site	Coordinates (UTM)	pH	Redox (mV)	Conductivity (mS/cm^2^)	Temperature (°C)	Dissolved O_2_ (ppm)
M01	0719806::4177816	2.2 ± 0.3	501.4 ± 22.2	22.7 ± 5.6	15.1 ± 6.6	6.9 ± 2.7
M02	0715837::4177837	2.6 ± 0.4	445.8 ± 44.9	6.2 ± 3.5	16.1 ± 6.8	7.5 ± 2.7
M03	0715838::4177796	2.4 ± 0.3	492.2 ± 19.2	13.1 ± 7.9	15.7 ± 7.4	7.4 ± 2.8
M04	0715859::4177815	4.7 ± 0.8	264.0 ± 112.7	3.9 ± 2.5	15.2 ± 5.1	8.4 ± 1.7
M05	0715842::4177739	2.6 ± 0.3	460.9 ± 25.3	9.47 ± 5.0	15.0 ± 6.1	8.6 ± 1.9
M06	0715692::4177662	2.1 ± 0.3	459.4 ± 12.2	27.6 ± 4.7	22.8 ± 1.5	3.9 ± 1.3
M07	0715498::4177618	1.9 ± 0.5	441.2 ± 20.4	47.8 ± 16.1	19.0 ± 2.2	3.9 ± 1.5
M08	0715300::4177585	2.5 ± 0.4	492.8 ± 43.1	12.8 ± 3.2	17.8 ± 0.9	3.1 ± 1.7
M09	0715347::4177577	2.2 ± 0.5	438.6 ± 24.9	29.6 ± .8	19.1 ± 5.6	4.7 ± 2.0
M10	0711361::4175713	2.4 ± 0.3	472.9 ± 81.1	4.1 ± 0.8	15.2 ± 5.5	9.2 ± 1.1
M11	0711361::4175713	2.6 ± 0.4	386.0 ± 31.9	6.7 ± 1.0	15.3 ± 0.7	1.9 ± 1.8
M12	0715022::4178096	2.6 ± 0.3	410.0 ± 29.9	7.0 ± 1.5	18.9 ± 6.8	8.3 ± 2.6
M13	0714775::4177710	2.6 ± 0.2	465.0 ± 36.1	5.9 ± 2.3	17.1 ± 6.1	3.3 ± 2.9
M14	0715227::4175634	2.6 ± 0.2	455.4 ± 47.0	9.7 ± 2.9	18.1 ± 7.8	6.9 ± 3.2
M15	0715227::4175634	2.5 ± 0.2	387.0 ± 22.4	16.6 ± 3.2	19.1 ± 1.9	3.7 ± 1.6
M16	0715031::4174228	2.6 ± 0.4	443.0 ± 16.1	8.9 ± 3.2	19.4 ± 7.6	8.3 ± 2.9
M18	0716325::4171834	2.6 ± 0.5	417.9 ± 22.1	9.0 ± 1.9	12.5 ± 4.7	9.4 ± 2.4
M19	0716303::4166707	2.8 ± 0.4	528.4 ± 73.4	4.1 ± 2.7	16.3 ± 6.1	9.5 ± 2.8
M21	0711505::4144569	2.7 ± 0.4	531.1 ± 69.9	2.9 ± 2.0	17.7 ± 6.5	10.0 ± 1.7

Values are the means of 19 measurements (**Supplementary Table S1**).

Water samples were taken from the surface of the river and the corresponding sediment samples from the bottom at the same spot (maximum depths of 20-30 cm). Río Tinto is a Mediterranean river with rather low flow at its origin all year round (samples M01 to M15). In the middle and lower parts of the river, the flow is higher, and the samples were taken from the riverside to avoid disturbing the sediment. Most of the samples were muddy, although a few of them, mostly in the origin part of the river, corresponded to wet sand gravel of very small size (maximum size 10-100 nm), mainly siliciclastic. The samples were collected in sterile 50-ml Falcon tubes and kept at 4°C until further processing in the laboratory. For each sample, water was filtered through 0.22 μm pore filters. These filters and the aliquots of the sediments were spread under sterile conditions on potato dextrose agar (PDA) plates supplemented with chloramphenicol (170 mg/ml) and incubated at room temperature until mycelia growth was observed. Visible mycelia were transferred to new fresh plates to obtain the axenic fungal isolates. Pure cultures were preserved in the Mycological Collection at the Unidad de Microbiología Aplicada (UMA) at the Centro de Biología Molecular Severo Ochoa (CSIC-UAM).

### DNA extraction, ITS rRNA amplification, and sequencing

Genomic DNA extraction from each of the 359 fungal isolates, ITS rDNA amplification, and sequencing, were performed according to the procedures described in Diáz et al. (2009). Briefly, a PowerSoil™ DNA kit (MO Bio Laboratories, Inc, USA) was used to extract the fungal DNA following the manufacturer´s specifications. A NanoDrop™ 1000 Spectrophotometer (NanoDrop Technologies Inc., USA) was used for DNA quantification. Primers ITS 1F (Gardes and Bruns, 1993) and ITS4 (White et al., 1990) were used to amplify the complete ITS regions. Polymerase chain reactions (PCR) were performed in 50µl volumes by mixing the template DNA (2 ng µl^-1^) with 1x PCR reaction buffer and 1.5 mM MgCl_2_, 0.25% (v/v) Tween 20, 5% dimethyl sulfoxide (v/v), 0.25 mM of each dNTP (GE Healthcare, UK), 2 µM of each primer, and 1.25 U Taq DNA Polymerase (Roche Diagnostics GmbH, Germany). Negative controls were included. Amplifications were done with a Perkin-Elmer Cetus 480 Thermal Cycler using the following protocol: a 2.5 min denaturation step at 94°C, followed by 40 cycles consisting of denaturation at 94°C for 30 s, primer annealing at 57°C for 30 s and extension at 72°C for 1.5 min, and a final extension at 72°C for 10 min. Amplification products were checked by ethidium bromide-stained 1% agarose gel electrophoresis, and purified with GFX™ PCR DNA and a Gel Band Purification kit (GE Healthcare, UK). Double-stranded PCR products were sequenced using the Taq Deoxy Terminator Cycle Sequencing kit (Perkin Elmer Applied Biosystems, USA) in an Applied Biosystems 373S DNA sequencer (Applied Biosystems, USA).

### Sequence data analyses

Forward and reverse sequences were assembled and edited using Geneious Pro 4.7.6 (Biomatters Ltd, New Zealand). Taxonomic labels were assigned through a similarity search in the NCBI’s nt collection in the GenBank database (updated 21^st^ August 2023) using the BLAST+ (Camacho et al., 2009) (v. 2.11.0) command line utility with default parameters. The *“-outfmt 6”* option was used to output a tabular format and include query sequence cover and scientific names. The results were filtered by >90% sequence identity and query cover and poorly annotated (i.e. uncultured fungus, fungal sp.) sequence matches were also filtered. For every sequence, the lowest common ancestor (LCA) over 90% of their remaining sequence matches was assigned with BASTA (Kahlke and Ralph, 2019). Additionally, the results were cross-checked with a search on the UNITE (Abarenkov et al., 2024) database (v9, updated 25^th^ July 2023) using Qiime2 (Bolyen et al., 2019) with 80% sequence identity and query cover. In this case, for every sequence, an LCA was assigned as the consensus of 51% over 10 matches (default Qiime2 parameters). To visualize the distance between sequences, a simple neighbor-joining tree (Saitou and Nei, 1987) was computed from a MUSCLE multiple sequence alignment using MEGA11 (Tamura et al., 2021) with default parameters. The sequences were uploaded to the European Nucleotide Archive (ENA) under the project name PRJEB65604.

### Non-metric multidimensional scaling

The incidence data of the fungal isolates across the sampling sites and time points was aggregated to the class level. Non-metric multidimensional scaling (NMDS) was calculated using Jaccard’s dissimilarity on class presence-absence data with the function ‘metaMDS’ in the vegan package of R (Oksanen et al., 2016). After 20 iterations, a minimum stress of 0.03 was achieved. Data were then represented by the first two ordination axes. Continuous environmental variables were fitted into the ordination (multiple regressions of environmental variables on the NMDS 1 and 2 axes, function ‘envfit’ in vegan). The significance of the regression was verified by a permutation test (999 permutations). The vector fit in the NMDS points to the direction of the steepest change in the environmental variable, implying a linear relationship. To avoid that problem and explore non-linear relationships between fungal community structure at the class level and environmental variables, a generalized additive model (GAM) regression for each individual variable was used to fit the surfaces into the NMDS (function ‘ordisurf’ in vegan package). Only statistically significant fits (p-value > 0.05) were projected into the ordination. Analysis was performed with the R (version 3.4.4) and the R CRAN vegan packages (v 2.4.6).

### Alpha diversity indexes

The proportion of species at each sampling site was first calculated following the formula P_i_ = N_i_/N_t_, where P_i_ is the proportion of a species i, N_i_ is the number of individuals of that species, and N_t_ is the total number of individuals in each sample. The Shannon index was then calculated by applying the equation ${\text{H}}^{'}=-\sum_{\text{i}=1}^{\text{S}}\left({\text{P}}_{\text{i}}\log{\text{P}}_{\text{i}}\right)$ . The Chao1 index was calculated according to the expression $\text{Chao}1={\text{ S}}_{\text{obs}}+\frac{{\text{F}}_{1}^{2}}{2\times {\text{F}}_{2}}$ , where S_obs_ is the total number of species observed, F_1_ is the number of singletons (species represented by a single individual), and F_2_ the number of doubletons (species represented by two individuals). In samples where no doubletons were detected, we applied a variation of the formula $\text{Chao}1^{*}={\text{ S}}_{\text{obs}}+\frac{F_{1}\times \left(F_{1}-1\right)}{2\times ({\text{F}}_{2}+1)}$ .

### Heavy metal tolerance assay and acidic pH tolerance

The sensitivities of 40 fungal strains to heavy metals, selected to represent the identified diversity and the different environmental conditions existing along the river, were determined by growth inhibition in a PDA plate assay at pH 2.8. The following heavy metals were used: Na_2_HAsO_4_.7H_2_O (As^5+^), CdSO_4_.8/3 H_2_O (Cd^2+^), CoSO_4_.7 H_2_O (Co^2+^), CuSO_4_.5 H_2_O (Cu^2+^), NiCl_2_.6 H_2_O (Ni^2+^), and Pb(NO_3_)_2_ (Pb^2+^). Stock metal solutions were prepared by dissolving their respective salts in double distilled water and sterilized by passing through a Millipore syringe filter with a 0.22 µm pore size. To estimate the maximum tolerable concentration (MTC), PDA broth medium was amended with increasing concentrations of the metal: 0,1mM, 1mM, 10mM, 50mM, 100mM, 200mM, 300mM, 500mM, 750mM, and 1000mM. Growth was measured after 2 weeks and monitored for an additional 6 weeks. To evaluate the fungal tolerance to the extreme conditions of Río Tinto, fungal strains were grown in liquid complete medium (CM) (5 g/L glucose, 5 g/L yeast extract, and 5 g/L malt extract) using water from sampling site M 19 with a pH of 2.6 and filtered through 0.22 μm pore filters.

### Transmission electron microscope

Sediment samples were fixed with 4% paraformaldehyde and 2% glutaraldehyde in 0.1 M phosphate buffer (pH 7.4) and washed with phosphate buffer (pH 7-4). Samples were treated with 1% osmium tetraoxide and 0.15% tannic acid in 0.1 M phosphate buffer (pH 7.4), stained with 2% uranyl acetate, washed between treatments, and dehydrated with a series of ethanol dilutions of 30%, 50%, 70%, 95%, and 3x 100% for 10 minutes at room temperature, with a final treatment of propylene oxide. Finally, samples were encapsidated and polymerized in BEEM capsules. Ultrathin sections were obtained with a Diatome diamond blade (Diatome-Us, Hatfield, PA, USA) in an Ultracut E (Leica,Wetzlar, Germany) ultramicrotome and collected on 200 mesh Cu grids. Sections were contrasted with uranyl acetate and lead citrate and examined in a JEM1010 transmission electron microscope (TEM) (JEOL, Tokyo, Japan) operating at 80 kV. EDX analyses were conducted using the STEM mode in a JEM 2010F (JEOL) equipped with an x-ray spectrometer operating at 200 kV.

## Results

### Sampling sites and physicochemical analyses

**Figure 3:**
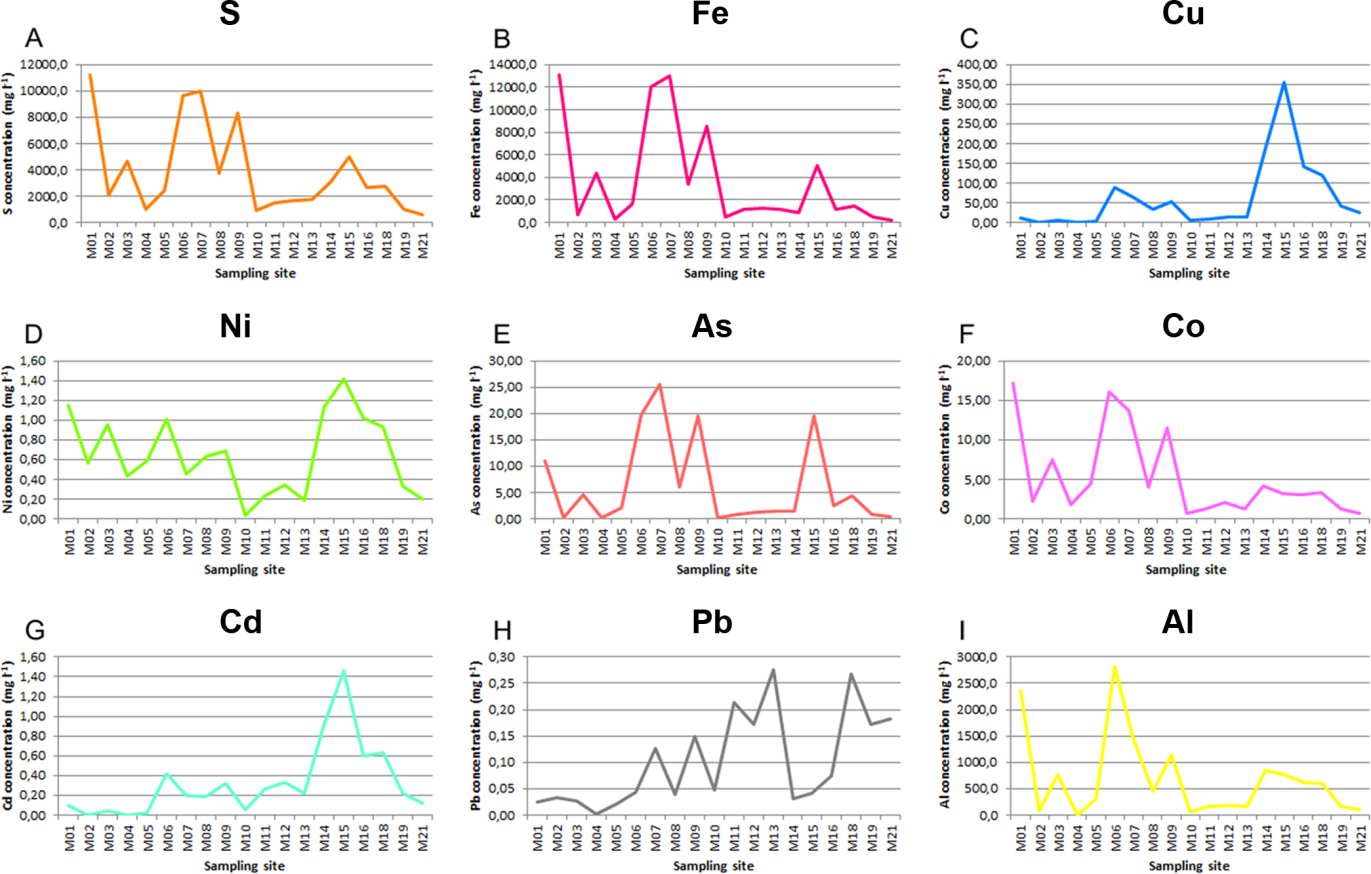
Average metal concentrations along the different sampling stations in the Tinto basin. **(A)** S, **(B)** Fe, **(C)** Cu, **(D)** Ni, **(E)** As, **(F)** Co, **(G)** Cd, **(H)** Pb, **(I)** Al. Represented data are the mean values of 19 measurements (**Supplementary Table S2**).

The sampling sites were selected using the water’s physicochemical characteristics to cover most of the acidic course of the river (**Figure 2**). The average pH at the 19 sampling areas and during the years 2009, 2010, and 2011 was 2.6 ± 0.6 (**Table 1**). The sampling stations with the lowest pH were M07, M06, and M01. These sampling stations were the ones with the highest conductivity values and with the highest sulfur and iron concentrations (**Figure 3**; **Table 1**; **Supplementary Tables S1**, **S2**). The highest iron concentrations averaged approximately 13,000 mg/L at sampling sites such as M01 or M07. Sulfur followed a similar pattern, with M02 and M07 being the sites that reached the highest levels. With respect to the rest of the heavy metals studied, the distribution patterns for As, Co, and Al were similar to those observed for both S and Fe, while as for Cu and Cd, they showed a different distribution pattern (**Figure 3**; **Supplementary Table S2**).

Regarding the water temperature, the average values fluctuated between 13.57 ± 3.50°C in winter to 24.48 ± 4.42°C in summer (**Supplementary Table S1**). The highest temperature was detected at sampling station M13 in the summer of 2010 (35.53°C), while the lowest temperature was recorded at M18 during the winter of 2009 (7.27°C). Although some sampling points showed a constant temperature throughout the different seasons of the year and over the different years, the following were those that came directly from the subsurface of the Iberian Pyrite Belt: M06, M07, and M15.

### Molecular characterization of fungal isolates from Río Tinto

To elucidate the degree of fungal diversity in this environment, we have distinguished between yeasts and filamentous fungi, and we have focused our work on the latter. A total of 359 fungal strains were isolated of which 214 were obtained from water samples and 145 from sediments (**Supplementary Table S3**). A higher number of isolates were obtained from sediment in the lower river course than from the more extreme upper sites. Thus, in sample M19, with less extreme pH and metal concentrations, 71.7% of the fungal isolates were obtained from sediment samples, while only 28.3% were isolated from water samples (**Supplementary Table S3**).

**Figure 4:**
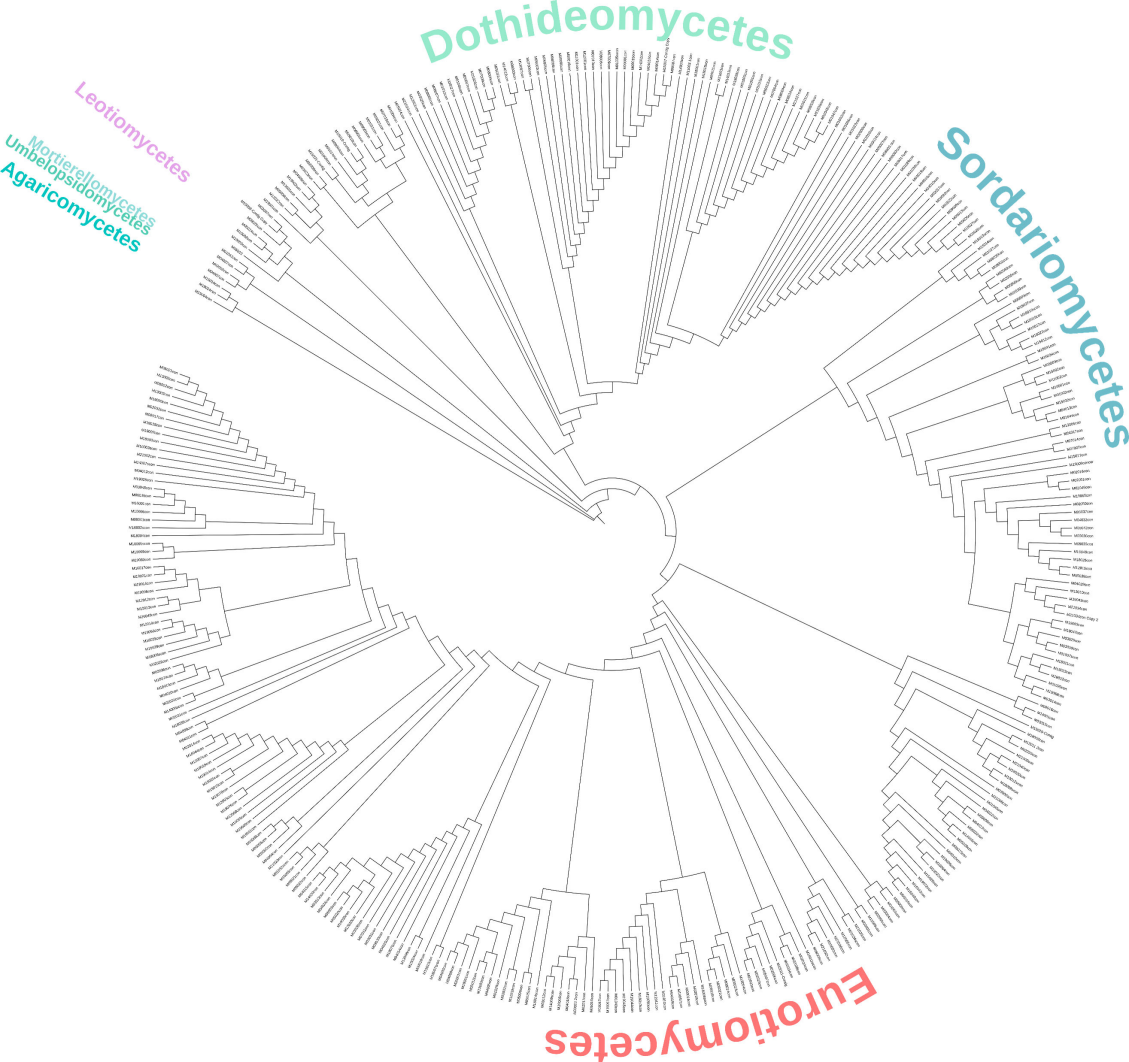
Neighbor-joining tree of the fungal isolates from Río Tinto.

A total of 359 ITS sequences were generated, and their analysis revealed an abundance of members of the Ascomycota phylum. Isolates from the Basidiomycota and Mucoromycota phyla accounted for only 2%. The most frequent group was Eurotiomycetes, with 51.3% of the fungal isolates, followed by 27.9% in the Dothideomycetes and 15.3% in the Sordariomycetes classes (**Figure 4**; **Supplementary Data Sheet S1**).

The isolates identified as members of the Eurotiomycetes class comprise 181 strains belonging to two families, Aspergillaceae and Trichocomaceae. Of these, 24 isolates clustered in the *Talaromyces* genus (Trichocomaceae family). The rest of the sequences clustered within the Aspergillaceae family, grouping with *Penicillium*, the most abundant genus along the river (34%), and the *Aspergillus* genera (7.8%) (**Figure 4**; **Table 2**; **Supplementary Data Sheet S1**).

**Table 2:** Percentage of the identified fungi at the different sampling sites along the Río Tinto.

Genus	Family	Order	Class	Phyla	%
*Penicillium*	Aspergillaceae	Eurotiales	Eurotiomycetes	Ascomycota	34.0
*Acidiella*	Teratosphaeriaceae	Micosphaerealles	Dothideomycetes	Ascomycota	20.9
*Trichoderma*	Hipocreaceae	Hypocreales	Sordariomycetes	Ascomycota	8.6
*Aspergillus*	Aspergillaceae	Eurotiales	Eurotiomycetes	Ascomycota	7.8
*Talaromyces*	Trichocomaceae	Eurotiales	Eurotiomycetes	Ascomycota	6.7
*Acidomyces*	Teratosphaeriaceae	Micosphaerealles	Dothiomycetes	Ascomycota	3.9
*Purpureocilium*	Ophiocordycipitaceae	Hypocreales	Sordariomycetes	Ascomycota	1.4
*Acidothrix*	Amplistromataceae	Hypocreales	Sordariomycetes	Ascomycota	1.1
*Fodinomyces*	Teratosphaeriaceae	Micosphaerealles	Dothiomycetes	Ascomycota	1.1
*Arcopilus*	Chaetomiaceae	Sordariales	Sordariomycetes	Ascomycota	0.8
*Dichotomopilus*	Chaetomiaceae	Sordariales	Sordariomycetes	Ascomycota	0.8
*Gleotinia*	Cordycipitaceae	Helotiales	Leotiomycetes	Ascomycota	0.8
*Penicillago*	Pleurostomataceae	Calosphaeriales	Sordariomycetes	Ascomycota	0.8
*Phialomyces*	Aspergillaceae	Eurotiales	Eurotiomycetes	Ascomycota	0.8
*Mucor*	Mucoraceae	Mucorales	Mucoromycetes	Mucoromycota	0.6
*Pholiota*	Strophariaceae	Agaricales	Agaricomycetes	Basidiomycota	0.6
*Pleurostoma*	Plerostomataceae	Calosphaeriales	Sordariomycetes	Ascomycota	0.6

Of the 100 strains that were placed within the Dothideomycetes class, 94 belonged to the order Mycosphaerealles, with the genus *Acidiella* being the second most abundant along the river (20.9%) (**Figure 4**; **Table 2**; **Supplementary Data Sheet S1**). The fungal isolates placed in the Sordariomycetes class were distributed mainly in three orders, the Hypocreales, Sordariales, and Calosphaeriales, with Hypocreales being the order with the highest number of isolates. The species related to the genus *Trichoderma* was the third most abundant in Río Tinto (8.6%), and the isolates from the genus *Purpureocillium* (1.4%) were grouped with a 100% bootstrap (**Figure 4**; **Table 2**; **Supplementary Data Sheet S1**).

Regarding the Basidiomycota and Mucoromycota phyla, eight strains grouped separately, four fungal isolates within the first phylum, and four within the second one. All the Basidiomycota strains grouped with the Agaricomycetes class, two within the Agaricales order showed high BLAST homology with members of the genus *Pholiota*. Of the four Mucoromycota isolates, two grouped in the Mucorales order, within the *Mucor* genus (**Table 2**; **Supplementary Data Sheet S1**).

### Río Tinto fungal ecology

**Figure 5:**
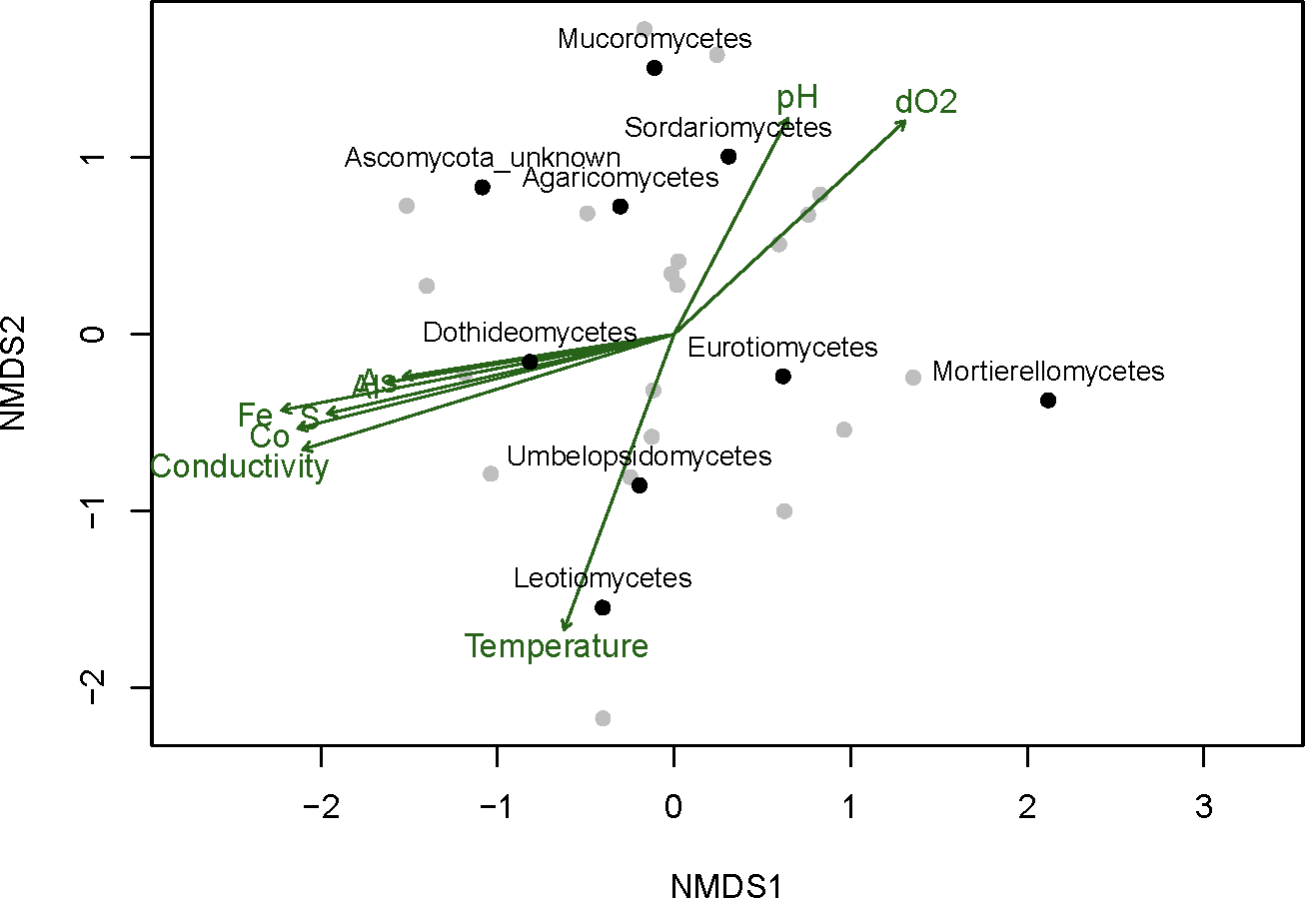
Non-metric multidimensional scaling (NMDS) based on Jaccard’s dissimilarity index of the presence or absence of fungal classes across all the samples. Samples and fungal classes are represented by grey and black dots respectively. Significant correlations of the NMDS axis with the continuous environmental variables (permutation test, *p-value* < 0.05) are indicated by the arrows pointing to the steepest change of the variable. dO2, dissolved O_2_.

To characterize the fungal ecology, samples were collected at different seasons in 2009, 2010, and part of 2011. The main source of fungal isolates was the water column. The distribution of the different isolates within the sampling stations showed that members of the Eurotiomycetes class dominate along most of the river sampling sites, except for the most extreme ones in the origin. At sampling site M07, all the isolates from this class were obtained from sediments, but at the M06 site, one of the most extreme parts of the river, no member of this class could be isolated there, and all fungal isolates from this site belonged to the Dothideomycetes class. The similarity in the distribution of the different classes across the sampled sites and its correlation with the environmental parameters was analyzed with NMDS (**Figure 5**; **Supplementary Figures S1**, **S2**; **Supplementary Table S4**). The results indicate that the presence of members of the Dothiodeomycetes class correlates with the sites where metal concentrations were higher. This fungal class dominates the most extreme areas of the river (**Supplementary Figures S1**, **S2**), since they were mainly isolated from water samples. It should be mentioned that this type of fungi was able to grow faster and more efficiently in Río Tinto water complete medium than in miliQ water complete medium when compared to other isolates from other classes.

The distribution profile of the Sordariomycetes class isolates along the river was similar to the Eurotiomycetes class. They were not present at all the sample stations. At sampling sites M06, M09, M10, and M14, we did not isolate any member of this fungal class. They were also preferentially isolated from sediment samples. In fact, this was the only source of isolates in the case of sampling stations M07, M12, and M16. However, at M08, a higher number of isolates was obtained from water samples.

The NMDS results also showed that the distribution of members of the Sordariomycetes, Eurotiomycetes, and Dothideomycetes classes at the different sampling sites was very distinct (**Figure 5**). Isolates of the Leotiomycetes class seemed to be related to sampling sites with higher temperatures. Mucoromycetes and Agaricomycetes isolates were obtained from sediment in the most extreme areas, while in the less extreme areas, isolates from these phyla were obtained from the water.

Furthermore, the alpha diversity indexes were obtained for the different sampling sites. The low Shannon values indicate that the richness of the different sampling sites was rather low, as expected for an extremely acidic environment. M15 was the most diverse and M10 the least diverse of the different sampling sites (**Supplementary Table S5**). The Chao 1 index values showed that more isolates were obtained from some sampling sites, such as M02, M04, M05, and M19 (**Supplementary Table S5**).

Our data showed there was less diversity in the areas with lower pH and higher heavy metal content such as M06 and M07. In the same way, the highest diversity was observed at sampling site M19 where the water’s physicochemical conditions were less extreme. The fungal isolates obtained at this site were mostly of the class Eurotiomycetes. No Dothideomycetes isolates were obtained at this sampling station. The obtained distribution pattern was maintained throughout the year without any observed seasonal change.

**Figure 6:**
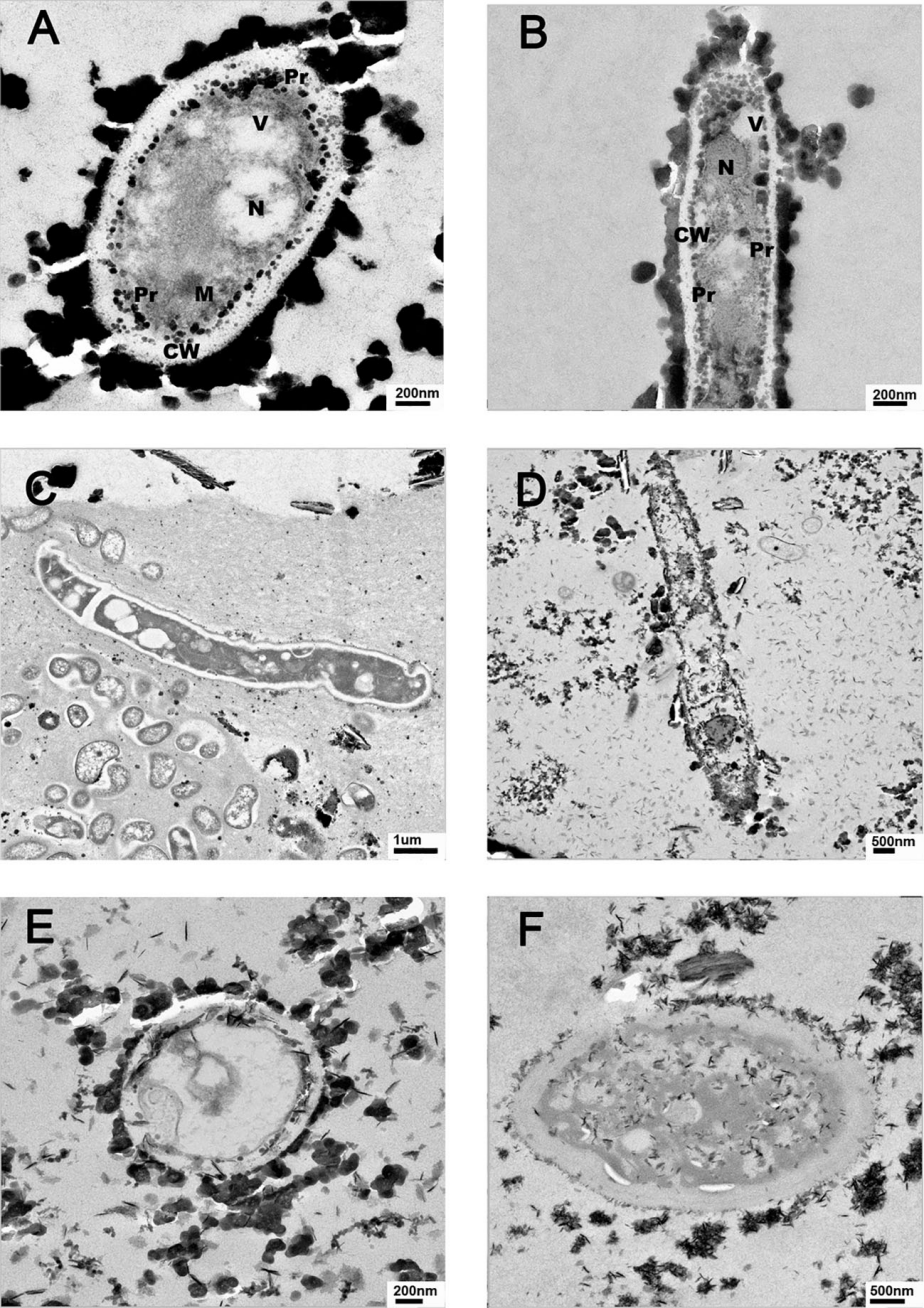
Representative TEM images of Río Tinto fungal cells detected in sediment from different sampling sites. **(A)** and **(B)** from M01, **(C)** M16, **(D)** M18, **(E)** M14, and **(F)**: M19.

Fungi and prokaryotes were observed in TEM images of all the examined sediments. The presence of a very thick cell walls and electron-dense structures associated externally or internally with the cell wall can be seen in the observed fungal structures (**Figure 6**).

### Heavy metal tolerance

To evaluate the tolerance of the isolates from Río Tinto to heavy metal-rich conditions, 40 isolates representing the different physicochemical conditions and the identified phylotypes along the Tinto basin were selected. The MTCs of the tested isolates are shown in **Table 3**. The most tolerant detected genera belonged to the *Penicillium* and *Purpureocillium* genera. The Basidiomycete strain M19004 did not show a high tolerance profile to heavy metals compared to other fungal isolates, but it is interesting to note that it was able to tolerate much higher levels of metals than those present in the area where it was isolated (M19). Although the tested isolates belonging to the genus *Acidiella* (Dothideomycetes) did not show high tolerance profiles to heavy metals, they were able to tolerate higher levels than those found at the sampling sites from which they were isolated.

**Table 3:** Maximum metal tolerance concentrations of selected fungal isolates to different heavy metals ordered by the location of the sampling sites.

Isolate	Closest taxa	As^5+^	Cd^2+^	Co^2+^	Cu^2+^	Ni^2+^	Pb^2+^
*M02001*	*Penicillium* sp.	3746	112.4	58.9	63.6	58.7	2072
*M02002*	*Mortierella* sp.	37460	1124.1	58.9	63.6	58.7	2072
*M02003*	*Penicillium raperi*	3746	11.2	58.9	63.6	58.7	2072
*M02004*	*Penicillago moldavica*	3746	112.4	589.3	63.6	58.7	2072
*M02006*	*Penicillium raperi*	3746	11.2	58.9	63.6	58.7	207.2
*M02040*	*Sporothrix* sp.	749.2	1124.1	58.9	635.5	58.7	2072
*M05001*	*Penicillium philippinense*	3746	11.2	58.9	63.6	58.7	2072
*M05002*	*Penicillium longicateatum*	74.9	11,2	589.3	63.6	58.7	2072
*M05003*	*Penicillium expansum*	74.9	11.2	58.9	63.6	58.7	2072
*M05004*	*Penicillium philippinense*	3746	11.2	58.9	63.6	58.7	207.2
*M05005*	*Pleurostoma richardsiae*	3746	11.2	58.9	63.6	58.7	207.2
*M05007*	*Penicillium raperi*	3746	11.2	58.9	635.5	58.7	2072
*M06001*	*Acidomyces acidophilus*	749.2	11.2	589.3	63.6	58.7	207.2
*M06002*	*Acidiella bohemica*	3746	1124.1	589.3	635.5	58.7	10360
*M06003*	*Fodinomyces uranophilus*	3746	1124.1	589.3	635.5	58.7	10360
*M06005*	*Acidiella bohemica*	3746	1124.1	589.3	635.5	58.7	10360
*M06006*	*Acidiella bohemica*	749.2	1124.1	589.3	635.5	58.7	10360
*M06007*	*Acidiella bohemica*	3746	1124.1	589.3	635.5	58.7	10360
*M06008*	*Acidiella bohemica*	3746	1124.1	589.3	635.5	58.7	10360
*M07014*	*Neonectria ditissima*	749.2	NC	58.9	635.5	5.9	2072
*M08002*	*Acidothrix acidophila*	3746	112.4	2946.5	63.6	586.9	207.2
*M09001*	*Acidiella bohemica*	74.9	NC	5.9	63.6	58.7	207.2
*M09002*	*Acidiella bohemica*	74.9	NC	5.9	63.6	58.7	207.2
*M09003*	*Acidomyces acidophilus*	749.2	11.2	58.9	63.6	58.7	207.2
*M09004*	*Acidomyces acidophilus*	3746	11.2	589.3	63.6	58.7	207.2
*M09005*	*Penicillium longicatenatum*	7492	11.2	589.3	635.5	58.7	2072
*M13002*	*Penicillium simplicissimum*	56190	5620.4	5893	6355	586.9	20720
*M13003*	*Penicillium philippinense*	37460	11.2	58.9	63.6	58.7	2072
*M13005*	*Penicillium simplicissimum*	56190	84310.5	5893	3177.5	5869	20720
*M13006*	*Cytospora austramontana*	NC	11.2	589.3	3177.5	586.9	2072
*M14001*	*Talaromyces* sp.	22476	11.2	58.9	635.5	5.9	207.2
*M15001*	*Purpureocillium lilacinum*	7492	84310.5	589.3	63550	586.9	2072
*M15002*	*Verticillium leptobactrum*	7492	84310.5	589.3	47662.5	5869	2072
*M15008*	*Gyoerffyella* sp.	3746	112.4	58.9	3177.5	58.7	2072
*M16001*	*Penicillium simplicissimum*	14984	11.2	2946.5	3177.5	2934.5	20720
*M18001*	*Acidiella bohemica*	74.9	NC	58.9	63.6	58.7	207.2
*M18002*	*Talaromyces borbonicus*	749.2	11.2	58.8	63.6	58.7	207.2
*M19004*	*Dichomitus squalens*	7.5	11.2	5.9	63.6	5.9	207.2
*M21001*	*Acidiella bohemica*	749.2	NC	5.9	635.5	586.9	10360
*M21024*	*Acidiella bohemica*	3746	11.2	5.9	3177.5	586.9	10360

Concentrations in mg/L, NC not measured.

Most of the tested isolates showed significant metal tolerance ranges, with some strains showing extremely high tolerances, such as *Purpureocillium lilacinum* M15001 to 1M (63.6 gr/L) of Cu^2+^and *Penicillium simplicissimum* M13005 to 750 mM (56.2 gr/L) of As^5^, 750 mM (84.3 gr/L) of Cd^2+^, 100 mM (5.9 gr/L) of Co^2+^, 100 mM (5.9 gr/L) of Ni^2+^, 100 mM (20 gr/L) of Pb^2+^, and 3.2 gr/L (50 mM) of Cu^2+^ (**Table 3**). The high tolerance of *Penicillium simplicissimum* M13005 to most of the tested metals and metalloids (poly-resistance) is noteworthy. Not all strains tolerated a certain level of metal from areas where the metal is in a high concentration. However, the strains observed to have the highest tolerance to Cu^2+^ and Cd^2+^ corresponded to strains isolated at sampling sites with high concentrations of these cations. For As^5+^ and Co^2+^, the more tolerant strains were isolated from areas in which the concentrations of these metals were not very high. The most metal poly-tolerant isolates were obtained from sampling site M15 (*Purpureocillium lilacinum* M15001 and *Gleotinia temulenta* M15002) and sampling site M13 (*Penicillium simplicissimum* isolates M13002 and M13005) (**Table 3**).

## Discussion

The main objective of this work was to describe the diversity of isolated filamentous fungi present in the water and sediment along the Río Tinto basin. There are relatively few reports on the diversity of eukaryotic acidophilic microorganisms in the literature as most of the research has been focused on prokaryotes due to their usefulness in biohydrometallurgy. In previous studies describing fungal diversity in the IPB, the identification of the isolates was done phenotypically (López-Archilla et al., 2001, 2004), or was centered on the study of yeast diversity (Gadanho et al., 2006). In this study, the ITS sequences of the different isolates were generated and analyzed to identify the fungal populations in the water and sediment of the river in different seasons over almost 3 years.

A decrease in the number of species at the sampling sites M06 and M07, which had the lowest pH (1.9) and highest heavy metal content, was observed. In most of the sampling sites where sufficient ferric iron and ferric hydroxides were present, the pH remained constant due to the buffer capacity of ferric iron (González-Toril et al., 2003). This acidic pH was also responsible for the high concentrations of heavy metals present in the river, which are several orders of magnitude higher than those reported in other local freshwater sources. For these reasons, Río Tinto is considered one the most extensive natural acidic ecosystems on Earth (Fernández-Remolar et al., 2003). Our data suggest that the fungal community found in the Tinto basin is generally independent of the season, with some influence of the temperature over the Leotiomycetes class, as shown by the NMDS analysis (**Figure 5**). Our data do not show the negative correlation between temperature and members of the *Penicillium* genera described by López-Archilla et al. in 2004.

The concentration of heavy metals, which is related with conductivity, has a strong influence on the Río Tinto fungal community. Conductivity values correlate with the metal concentrations and vary among seasons depending on the rainfall, thus, in rainy seasons the observed conductivity was lower than in the seasons when rain was scarce. In any case, the high levels of soluble heavy metal cations found along the 92-km river are quite remarkable. At sampling sites with high conductivity and elevated metal concentrations, the predominant fungal populations belonged to the Dothideomycetes class, with *Acidiella* being the most represented genus. This pattern was observed at sites M01, M06, M07, M09, and M15, which had the highest concentrations of Fe, As, Co, Ni, Cu, and Cd, coupled with the lowest pH values, making them the most extreme sites in the Tinto Basin. Conversely, at less extreme sites with lower metal concentrations and conductivity, the predominant fungi were members of the Eurotiomycetes class. These results are consistent with previous findings (López-Archilla et al., 2004) which reported a significant increase in dematiaceous fungi (Dothideomycetes) at lower pH values and high heavy metal concentrations, while *Penicillium* (Eurotiomycetes) showed the opposite correlation.

Many of the phylotypes identified in this study are related to acidophilic microorganisms isolated from mine sites and metal-contaminated soils (Anahid et al., 2011; Liaquat et al., 2020; Mohammadian et al., 2017; Ou et al., 2021), and they have previously been described in Río Tinto (López-Archilla et al., 2004).Thus, our cladogram confirms that the highest number of isolates corresponded to the Euromycetes class (49.3%), with *Penicillium* being the most represented genus (34%), followed by the Dothideomycetes class (25.9%), of which 20.9% were members of the genus *Acidiella*. This, in addition to the fact that all the isolated *Penicillium* and *Acidiella* strains were able to grow in the extreme conditions of the medium amended with Río Tinto water, highlights the adaptability of these genera to tolerate extreme conditions. Among the isolates of the *Penicillium* genus, we found significant tolerance rates to the tested metals, reaching levels of 56.2 gr/L for As^5+^, 84.3 gr/L for Cd^2+^, 5.9 gr/L for Co^2+^, 20 gr/L for Pb^2+^, 5.9 gr/L for Ni^2+^, and 3.2 gr/L for Cu^2+^. To the best of our knowledge, the observed metal tolerance exhibited by some of the Río Tinto fungal isolates is the highest described thus far for fungi, especially for Cu^2+^ and As^5+^ (Anahid et al., 2011; Medina-Armijo et al., 2024; Mohammadian et al., 2017; Sing and Narzary, 2023). Some of the isolates encompassed multiple strains of the same species, such as for *Acidiella bohemica* or *Penicillium simplicissimum*. The comparison of the metal tolerance among these strains showed wide interspecific variability, which has been observed for many other fungal isolates (Medina-Armijo et al., 2024; Sing and Narzary, 2023), and which requires further study to understand the metabolic potential and adaptation mechanisms of fungi in AMD environments (Ou et al., 2021).

In accordance with these results, several authors have isolated different *Penicillium* species from environments with high heavy metal contents (Glukhova et al., 2018; López-Archilla et al., 2004) that are able to grow even at copper sulfate concentrations close to saturation (Okamoto and Fuwa, 1974), as in our case. This was not the case for the tested *Acidocella* isolates which, although they were able to grow in the metal concentrations of the sampling sites in which they were isolated, they did not show the high metal tolerance exhibited by some members of the *Penicillium* genus. Furthermore, poly-tolerance to heavy metals has been detected in other selected isolates, such as *Verticillium leptobactrum* M15002.

We found that Cd^2+^ was the most toxic heavy metal tested, which agrees with Itoh et al. (1975), followed by Ni^2+^, Cu^2+^, Co^2+^, Pb^2+^, and As^5+^ in decreasing order of toxicity. It is reasonable to postulate that the observed tolerance of an isolate to a given metal must be related to the concentration of this metal at the sampling site from which it was isolated, but this was not true in all cases. Thus, in most cases, no direct correlation was found between the metal tolerated by the fungi and the metal content at the sampling site (**Figure 5**).

The strains from the Dothideomycetes class are characterized by the production of large amounts of melanin. Melanin is a fungal pigment that plays an important role in fungal survival in response to environmental stress, providing efficient metal binding sites. Fungal melanin can bind significant amounts of heavy metals (Cordero et al., 2017; Gadd and de Rome, 1988; Medina-Armijo et al., 2024). Although strains of this class did not reach the tolerance levels achieved by the Eurotiomycetes isolates, all of them were able to grow more efficiently in medium amended with Río Tinto water. Furthermore, in all cases, they were able to tolerate metal concentrations that exceeded those found at the sampling sites. They have been described as slow-growing melanized colonizers of rock surfaces in extreme environments (Gadd, 2007; Gueidan et al., 2011; Ruibal et al., 2009; Sterflinger, 2000) and are able to tolerate low nutrient availability, fluctuations in temperature, and high solar radiation intensity, among other extreme conditions (Gorbushina et al., 2008). These characteristics could explain why members of this group of fungi are able to grow in environments with high concentrations of heavy metals such as Río Tinto. Moreover, due to their survival abilities, they have garnered the interest of astrobiologists (Gorbushina, 2003; Onofri et al., 2008). Thus, due to the correlation of this fungal class with pH and the heavy metal concentration, we also suggest in accordance with López-Archilla et al. (2004) that these fungi are active members in this acidic extreme environment.

Although members of the Sordariomycetes class are the least abundant class of Ascomycota detected in Río Tinto, they are present in most of the less extreme parts of the river. As shown in **Supplementary Figure S1**, they were preferentially isolated in sediment. Members of this class have been also isolated from habitats with high concentrations of heavy metals (Daghino et al., 2008; Oggerin et al., 2013; Tatsuyama et al., 1975), and some of the Río Tinto isolates have shown an amazing ability to tolerate high concentrations of certain metals, such as the isolate M15001, identified as a *Purpureocillium lilacinum*, reaching levels of up to 63.5 gr/L (1M) of Cu^2+^, 84.3 gr/L (750mM) of Cd^2+^, and 7.5 gr/L (100mM) of As^5+^. Among them are found the most highly tolerant strains resistant to several metals simultaneously (poly-tolerance). The mechanisms used by different fungal isolates from Río Tinto to withstand the presence of different metals are currently under study. Preliminary results showed that in certain cases, melanin synthesis is involved, which may provide the means for metal binding, enhancing their metal tolerance (Gadd and de Rome, 1988; Medina-Armijo et al., 2024). In other isolates, heavy metal ions are compartmentalized inside the cell in vacuoles accumulating high concentrations of metals, as has been shown in other fungal strains (Durán et al., 1999; Gadd, 1990).

With regard to the fungal isolates within the Helotiales order, members of this order have been isolated previously from mine streams and soils (Miersch et al., 2001; Sharples et al., 2001). Some species of *Mortierella* and *Mucor* were isolated from acidic soils with high levels of heavy metals (e.g. Kendrick, 1962; Miersch et al., 2001; Seifert, 2008), therefore the occurrence of these fungi in an ecosystem such as Río Tinto is not surprising. One of these isolates, M02002, showed a high level of tolerance to As^5+^ since it was able to grow in the presence of 37.5 gr/L (500 mM) of this metalloid.

Although some authors have shown that the frequency of metal-tolerant microorganisms is associated with the presence of toxic metals in the environment from which they were isolated (Pennanen et al., 1996), in our case, no correlation was found between the metal content of the sampling sites and their metal tolerance, except for sample M15 isolates. This is an area with the highest levels of Cu and Cd in the whole river, and a large proportion of the fungal strains that were isolated from this area showed remarkable tolerance to Cu^2+^. In a previous study, we found that in some cases this ability to grow in the presence of high heavy metal concentrations required no adaptation period. Some fungal isolates were able to grow at the maximum metal tolerance concentrations, while other fungal isolates required a previous adaptation to these conditions, i.e., an induction exposing the isolate to increasing concentrations of heavy metals (Durán et al., 1999). In these conditions, fungal survival and growth is possible due to a range of different strategies such as cell wall thickening, metal sorption to cell walls, intracellular compartmentalization, or biomineralization, amongst others (Brown and Hall, 1990; Gadd, 2008).

Studies in our laboratory on fungal strains isolated from Río Tinto grown in the presence of different concentrations of heavy metals showed in most cases a thickening of the cell walls. However, depending on the tested heavy metals, the strategies used by the fungi were different. In some cases, internal accumulation of metals in the vesicles was observed (Durán et al., 1999). In others, electrodense structures associated with the external cell wall were formed. In fact, a study performed with *Purpureocillium lilacinum* M15001 grown in the presence of Cu^2+^ demonstrated a thickening of the fungal cell wall using TEM and detected the overtranslation of several proteins involved in the chitin synthesis pathway (Oggerin et al., 2015), clathrin and snare, and proteins involved in vesicle synthesis (Kirchhausen, 2000) using proteomic analysis. Clathrin-coated vesicles are involved in many cellular processes such as the regulation of osmotic pressure, synthesis of large vacuoles, or cell wall synthesis (de León et al., 2013). The observation of large vacuoles containing large amounts of Cu using TEM, strongly suggests that this mechanism must be in operation in this isolate to overcome the extremely high concentration of Cu^2+^ present in the growth medium. The TEM analysis performed with samples of river sediment revealed the presence of fungal structures at all sampling sites. As shown in **Figure 6**, some representative samples show the presence of fungal cells with very thick cell walls.

It is well established that fungi are actively involved in the biochemical cycles through mineral weathering (Gadd et al., 2011). Oggerin and collaborators (Oggerin et al., 2013) were able to verify that some fungi play a role in the Río Tinto geochemical cycles through biomineralization processes, with minerals nucleated on the fungal cell walls. Preliminary studies with *Purpureocillium lilacinum* (isolate M15001) showed that fungal cells were actively involved in biomineralization processes in natural sediment from Río Tinto through the formation of lepidocrocite, a mineral that, according to the physicochemical characteristics of the environment, should not be present (Oggerin et al., 2013, 2016).

In Río Tinto, it is known that most of the biomass can be found in biofilms, which are composed, mainly, of filamentous fungi and algae with different types of trapped bacteria (Aguilera et al., 2007a, b; Durán et al., 1999; López-Archilla et al., 2001). Some unpublished observations strongly suggest that fungi are the primary colonizers of these biofilms. The results show that many of the isolated fungal strains come from sediment. These filamentous fungi may contribute to the attachment of the biofilm to sediment particles and may confer a stable structure to them (Baker and Banfield, 2003; Hujslová et al., 2019). Biofilms might also protect from the high concentrations of heavy metals in solution since the main components of these biofilms are extracellular polymeric substances (EPS) (Aguilera et al., 2007a) characterized by their ability to bind cations due to the presence of negatively charged groups (Brown and Lester, 1979; Marqués et al., 1990; Scott and Palmer, 1988). This property could explain the presence of some isolates that are extremely sensitive to the heavy metals present in the water column.

The presence of fungi in the sediment proves that they are not simple contaminants or sporulation-resistant forms gathered in the river, but an important part of the ecosystem, developing mechanisms to overcome the high concentration of heavy metals present in the environment. Studies on acidophilic fungi are not only useful for our understanding of their role in the geomicrobiology of the environment in which they develop, but additionally allow us to explore their potential for biotechnological applications (Hujslová et al., 2019; Javaid et al., 2019). Recently, many of the fungal isolates described in this work have been used to identify enzymatic activities of interest in the production of biofuels (Daddaoua et al., 2023).

## Conclusions

The molecular characterization of 359 fungal isolates obtained from 19 sampling sites along the course of the Río Tinto basin allowed the seasonal diversity and its correlation with the variable physicochemical conditions existing in the river to be studied. The taxonomic affiliation using complete ITS sequences showed a high degree of diversity, with members of the Ascomycota phylum being the most abundant, while members of the Basidiomycota and Mucoromycota phyla accounted for only 2% of the identified fungal diversity. Of the identified Ascomycota, 51.3% were clustered within the Eurotiomycetes class with members of the *Penicillium* genus among the most abundant. Furthermore, 27.9% were clustered within Dothideomycetes, of which 20.9% were identified as members of the genus *Acidiella*, and 15.3% within Sordariomycetes, with *Trichoderma* being the most abundant genus (8.6%). Members of the Dothideomycetes class dominated the most extreme areas of the river. Of the fungal isolates, *Penicillium simplicissimum* M13005 stands out due to its ability to grow in the presence of 750 mM of As^5+^and Cd^2+^; 100 mM of Co^2+^, Ni^2+^, and Pb2+; and 50 mM of Cu^2+^, making it the most metal poly-tolerant fungi described thus far. The isolate *Purpureocillium lilacinum* M15001 was able to grow in the presence of 1 M of Cu^2+^. Different strains of *Penicillium simplicissimum* and *Purpureocilium lilacinum* showed important interspecific metal tolerance differences which require further study.

## Data Availability

The datasets presented in this study can be found in online repositories. The names of the repository/repositories and accession number(s) can be found in the article/**Supplementary Material**.

## Supplementary material

Supplementary Table 1 Physicochemical parameters of Río Tinto water during the 2009-2011 study period.

Supplementary Data Sheet 1 Taxonomic affiliation of the fungal isolates using the NCBI database.

Supplementary Figure 1 NMDS analysis with fitted surfaces for selected individual environmental variables. Significant correlations of all variables with the NMDS axes are presented. **(A)** temperature (°C), **(B)** pH, **(C)** conductivity (mS/cm^2^), and **(D)** dissolved oxygen (ppm). The blue lines connect points with the same value for the corresponding variable.

Supplementary Figure 2 NMDS analysis with fitted surfaces for selected individual metal variables in mg/L. Significant correlations of all variables with the NMDS axes are presented. **(A)** Aluminum, **(B)** Arsenic, **(C)** Sulfur, **(D)** Cobalt, **(E)** Iron, and **(F)** conductivity (same panel than Supplementary Figure S2C, included for comparison with the metals). The blue lines connect points with the same value for the corresponding variable.
