# Supplementary material for: Metal tolerance of Río Tinto fungi
Source: Front Fungal Biol. 2024 Oct 16;5:1446674. doi: 10.3389/ffunb.2024.1446674 (PMC11521807; doi:10.3389/ffunb.2024.1446674)
Supplement: Supplementary Table 1 — Physicochemical parameters of Río Tinto water during the 2009-2011 study period. [file Table1.docx]

|  |  |  |  |  |  |
| --- | --- | --- | --- | --- | --- |
| **2009** | | | | | |
| **Samplig site** | **pH** | **Redox (mV)** | **Conductivity (mS/cm2)** | **Temperature (ºC)** | **Dissolved O_2_ (ppm)** |
| M01 | 2,4±0,22 | 478,3±12,8 | 28,0±1,8 | 17,1±7,3 | 4,6±1,5 |
| M02 | 2,9±0,48 | 412,1±51,7 | 8,7±1,3 | 18,1±6,2 | 6,1±1,5 |
| M03 | 2,4±0,08 | 477,2±9,5 | 20,5±5,9 | 18,0±7,9 | 5,7±1,8 |
| M04 | 4,3±0,65 | 242,4±87,2 | 5,4±2,2 | 16,6±5,0 | 6,6±2,4 |
| M05 | 2,7±0,09 | 466,1±11,6 | 12,6±2,9 | 16,4±5,8 | 7,9±0,8 |
| M06 | 2,3±0,21 | 454,7±14,9 | 31,5±3,9 | 21,9±1,9 | 4,7±0,4 |
| M07 | 2,1±0,49 | 423,1±19,6 | 59,9±11,5 | 17,7±2,4 | 3,1±1,1 |
| M08 | 2,7±0,46 | 462,0±26,8 | 15,8±1,2 | 17,4±1,2 | 3,3±1,1 |
| M09 | 2,3±0,56 | 424,2±33,7 | 37,7±6,2 | 18,4±4,6 | 4,8±0,3 |
| M10 | 2,5±0,16 | 497,7±74,0 | 5,2±0,2 | 18,0±7,2 | 7,5±1,4 |
| M11 | 2,9±0,35 | 379,0±64,1 | 7,5±1,7 | 15,9±0,1 | 5,7±2,6 |
| M12 | 2,7±0,13 | 383,8±20,0 | 8,5±0,3 | 21,0±4,8 | 6,5±1,7 |
| M13 | 2,6±0,13 | 449,1±24,2 | 8,3±0,1 | 19,5±8,3 | 6,7±2,3 |
| M14 | 2,6±0,09 | 483,6±5,4 | 12,3±0,7 | 18,9±6,6 | 5,2±1,1 |
| M15 | 2,5±0,19 | 373,2±17,0 | 19,7±1,6 | 20,1±0,9 | 5,0±2,8 |
| M16 | 2,6±0,07 | 436,0±8,3 | 11,5±1,5 | 19,7±7,8 | 5,8±3,4 |
| M18 | 2,9±0,32 | 405,3±23,0 | 10,0±0,9 | 12,4±5,0 | 7,8±1,1 |
| M19 | 2,7±0,07 | 558,0±44,8 | 5,9±2,4 | 17,6±6,2 | 8,7±2,8 |
| M21 | 2,5±0,11 | 584,0±46,1 | 4,7±1,4 | 19,5±5,9 | 9,0±2,58 |
|  |  |  |  |  |  |
|  | **pH** | **Redox (mV)** | **Conductivity (mS/cm2)** | **Temperature (ºC)** | **Dissolved O_2_ (ppm)** |
| 2009 average | 2,7±0,3 | 441,3±31,3 | 16,5±2,5 | 18,1±5,0 | 6,0±1,7 |
|  |  |  |  |  |  |
| **2010** | | | | | |
| **Samplig site** | **pH** | **Redox (mV)** | **Conductivity (mS/cm2)** | **Temperature (ºC)** | **Dissolved O_2_ (ppm)** |
| M01 | 1,9±0,1 | 518,7±16,6 | 20,0±4,9 | 16,6±7,9 | 7,2±3,7 |
| M02 | 2,5±0,2 | 465,3±28,2 | 5,4±1,0 | 16,8±8,6 | 7,0±4,0 |
| M03 | 2,2±0,3 | 500,9±8,7 | 10,4±8,0 | 16,0±8,8 | 7,9±3,7 |
| M04 | 5,0±0,7 | 253,3±93,4 | 3,5±2,6 | 15,7±6,3 | 8,7±1,0 |
| M05 | 2,3±0,1 | 470,3±1,9 | 8,7±5,4 | 15,9±7,7 | 8,5±2,5 |
| M06 | 1,9±0,2 | 468,0±7,8 | 26,3±1,7 | 23,5±1,4 | 3,2±1,2 |
| M07 | 1,6±0,2 | 457,6±6,9 | 44,3±12,2 | 20,4±1,1 | 3,1±1,1 |
| M08 | 2,2±0,4 | 514,3±53,7 | 11,6±1,5 | 18,2±0,8 | 1,8±0,1 |
| M09 | 1,8±0,2 | 454,2±15,4 | 26,6±7,6 | 20,3±9,0 | 4,2±3,0 |
| M10 | 2,2±0,0 | 458,3±110,3 | 3,7±0,2 | 16,1±6,6 | 9,4±0,9 |
| M11 | 2,3±0,2 | 399,7±6,0 | 6,2±0,6 | 15,0±0,7 | 1,0±0,1 |
| M12 | 2,5±0,3 | 436,4±20,7 | 6,1±0,6 | 18,5±10,6 | 8,4±3,7 |
| M13 | 2,4±0,1 | 493,8±33,9 | 4,1±1,5 | 21,1±12,5 | 4,2±5,4 |
| M14 | 2,4±0,0 | 435,0±72,0 | 8,7±2,2 | 19,7±11,3 | 8,4±1,0 |
| M15 | 2,3±0,1 | 406,2±19,6 | 14,3±2,8 | 18,5±2,9 | 3,1±0,4 |
| M16 | 2,4±0,2 | 458,3±6,8 | 7,8±2,6 | 21,3±10,0 | 8,1±3,5 |
| M18 | 2,1±0,1 | 434,0±20,4 | 9,2±0,8 | 13,5±6,4 | 9,1±3,3 |
| M19 | 2,6±0,2 | 533,1±95,2 | 3,2±2,5 | 17,2±8,0 | 8,6±2,2 |
| M21 | 2,7±0,4 | 513,3±62,7 | 1,93±1,4 | 18,4±2,0 | 9,8±2,2 |
|  |  |  |  |  |  |
|  | **pH** | **Redox (mV)** | **Conductivity (mS/cm2)** | **Temperature (ºC)** | **Dissolved O_2_ (ppm)** |
| 2010 average | 2,4±0,7 | 456,4±61,7 | 11,7±10,7 | 18,0±2,5 | 6,4±2,9 |
|  |  |  |  |  |  |
| **2011** | | | | | |
| **Samplig site** | **pH** | **Redox (mV)** | **Conductivity (mS/cm2)** | **Temperature (ºC)** | **Dissolved O_2_ (ppm)** |
| M01 | 2,6±0,0 | 508,4±0,5 | 16,2±0,1 | 7,7±0,1 | 9,9±0,1 |
| M02 | 2,9±0,0 | 488,3±1,1 | 0,9±0,0 | 7,9±0,0 | 10,4±0,0 |
| M03 | 2,76±0,0 | 511,0±0,1 | 2,4±0,3 | 7,9±0,0 | 10,1±0,1 |
| M04 | 5,1±0,0 | 143,4±0,4 | 0,4±0,0 | 9,3±0,0 | 10,0±0,0 |
| M05 | 3,0±0,0 | 415,3±0,6 | 2,2±0,1 | 7,9±0,1 | 10,0±0,0 |
| M06 | 2,5±0,0 | 448,0±0,3 | 19,7±0,0 | 23,5±0,0 |  |
| M07 | 2,5±0,0 | 446,0±2,4 | 22,3±0,1 | 18,7±0,0 | 7,4±1,7 |
| M08 | 3,0±0,0 | 520,8±1,9 | 7,4±0,0 | 17,6±0,0 | 6,5±0,1 |
| M09 | 2,7±0,0 | 434,9±0,8 | 14,5±0,2 | 17,7±0,1 | 6,2±0,1 |
| M10 | 3,0±0,0 | 532,0±0,0 | 3,5±0,0 | 9,6±0,0 | 10,0±0,0 |
| M11 | 2,9±0,0 | 359,4±0,8 | 6,2±0,0 | 15,1±0,0 | 12,1±1,0 |
| M12 | 3,0±0,0 | 409,0±0,9 | 4,9±0,1 | 13,6±0,0 | 10,2±0,1 |
| M13 | 3,0±0,0 | 434,2±2,1 | 4,5±0,0 | 10,5±0,0 | 2,2±0,0 |
| M14 | 3,1±0,0 | 432,4±11,1 | 4,9±0,0 | 11,2±0,1 | 10,4±0,4 |
| M15 | 2,9±0,0 | 370,9±0,8 | 14,6±0,0 | 18,2±0,0 | 3,4±0,1 |
| M16 | 3,4±0,0 | 417,8±2,5 | 4,0±0,0 | 12,6±0,0 | 11,1±0,4 |
| M18 | 3,4±0,1 | 407,8±3,0 | 5,2±0,2 | 9,4±0,0 | 12,0±0,5 |
| M19 | 3,6±0,0 | 425,4±0,6 | 0,6±0,0 | 10,0±0,0 | 11,4±0,2 |
| M21 | 3,4±0,0 | 425,8±0,3 | 0,5±0,0 | 10,1±0,0 | 11,2±0,3 |
|  |  |  |  |  |  |
