# Supplementary figures and images for: Metal tolerance of Río Tinto fungi
Source: Front Fungal Biol. 2024 Oct 16;5:1446674. doi: 10.3389/ffunb.2024.1446674 (PMC11521807; doi:10.3389/ffunb.2024.1446674)

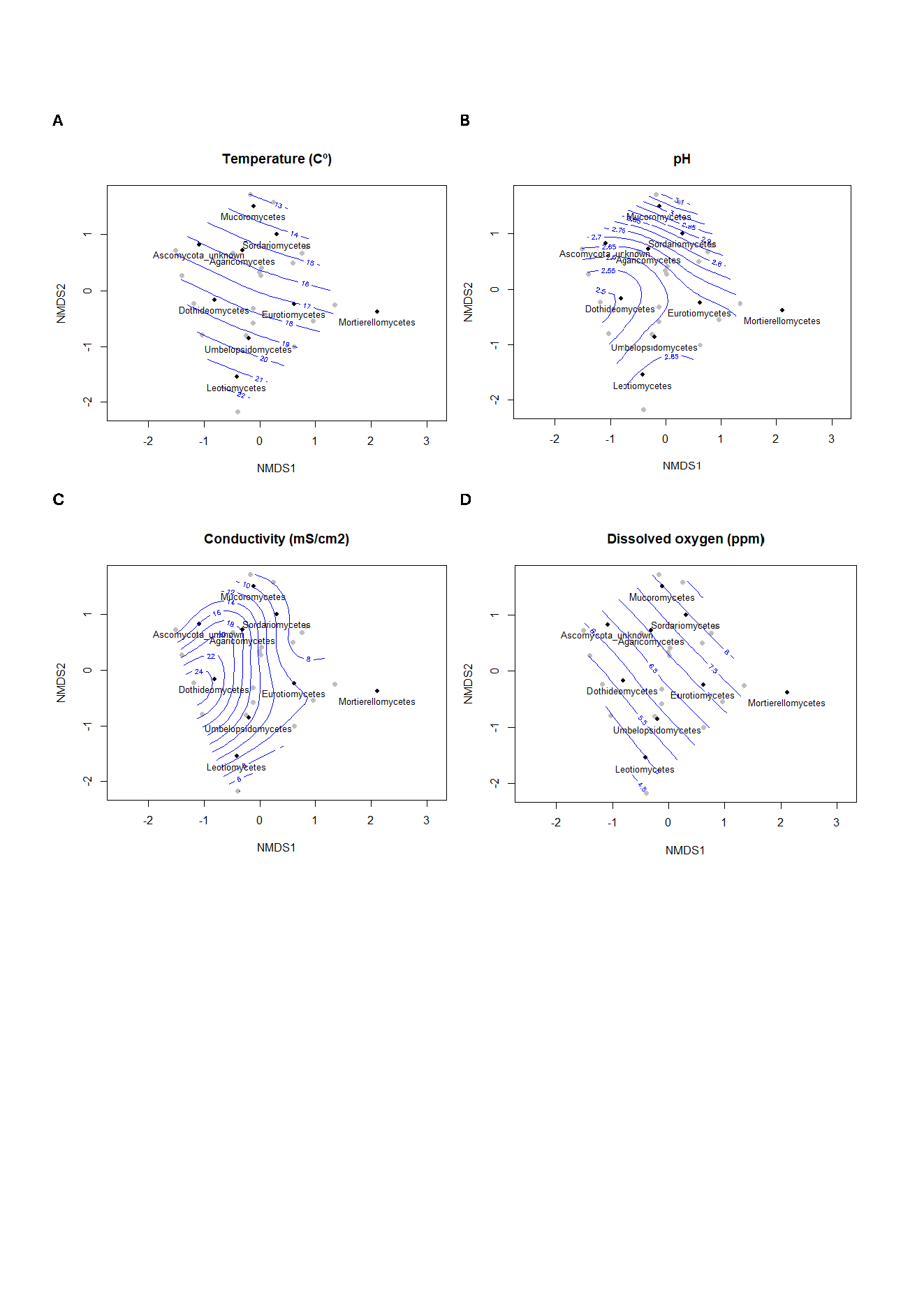

Supplement: Supplementary Figure 1 — NMDS analysis with fitted surfaces for selected individual environmental variables. Significant correlations of all variables with the NMDS axes are presented. (A) temperature (°C), (B) pH, (C) conductivity (mS/cm2), and (D) dissolved oxygen (ppm). The blue lines connect points with the same value for the corresponding variable. [file Image1.tif]

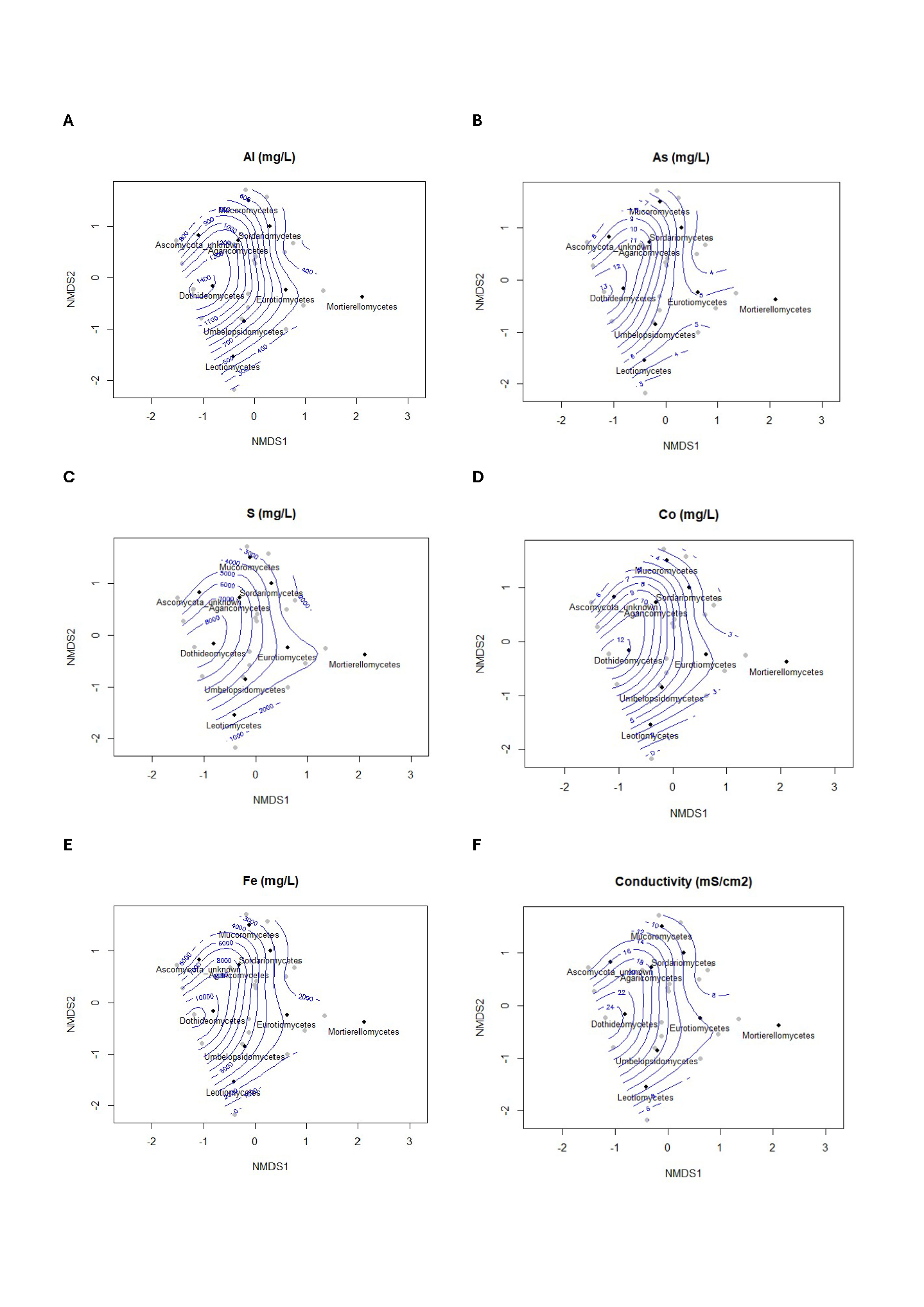

Supplement: Supplementary Figure 2 — NMDS analysis with fitted surfaces for selected individual metal variables in mg/L. Significant correlations of all variables with the NMDS axes are presented. (A) Aluminum, (B) Arsenic, (C) Sulfur, (D) Cobalt, (E) Iron, and (F) conductivity (same panel than Supplementary Figure S2C, included for comparison with the metals). The blue lines connect points with the same value for the corresponding variable. [file Image2.tif]
