# Supplementary material for: Metal tolerance of Río Tinto fungi
Source: Front Fungal Biol. 2024 Oct 16;5:1446674. doi: 10.3389/ffunb.2024.1446674 (PMC11521807; doi:10.3389/ffunb.2024.1446674)
Supplement: Supplementary file 6 [file Table3.docx]

**Table S3.** **List of the fungal isolates showing the date, the sampling site, and from where they were isolated.**

| **Strain** | **Sampling site** | **From** | **Sampling date** |
| --- | --- | --- | --- |
| M01001 | M01 | Water | March 2009 |
| M01002 | M01 | Water | March 2009 |
| M01003 | M01 | Water | March 2009 |
| M01004 | M01 | Water | June 2009 |
| M01005 | M01 | Water | June 2009 |
| M01006 | M01 | Water | June 2009 |
| M01009 | M01 | Water | June 2009 |
| M01010 | M01 | Water | December 2009 |
| M01011 | M01 | Water | December 2009 |
| M01012 | M01 | Water | December 2009 |
| M01014 | M01 | Water | December 2009 |
| M01015 | M01 | Water | December 2009 |
| M01016 | M01 | Water | December 2009 |
| M01022 | M01 | Water | December 2009 |
| M01026 | M01 | Sediment | June 2010 |
| M01027 | M01 | Sediment | June 2010 |
| M01029 | M01 | Sediment | November 2010 |
| M01030 | M01 | Sediment | November 2010 |
| M01032 | M01 | Sediment | November 2010 |
| M01034 | M01 | Sediment | November 2010 |
| M01035 | M01 | Sediment | November 2010 |
| M01037 | M01 | Sediment | November 2010 |
| M01038 | M01 | Sediment | March 2011 |
| M01042 | M01 | Sediment | March 2011 |
| M01043 | M01 | Sediment | March 2011 |
| M01044 | M01 | Sediment | March 2011 |
| M01045 | M01 | Sediment | March 2011 |
| M01046 | M01 | Sediment | March 2011 |
| M01047 | M01 | Sediment | March 2011 |
| M02001 | M02 | Water | March 2009 |
| M02002 | M02 | Water | March 2009 |
| M02003 | M02 | Water | March 2009 |
| M02004 | M02 | Water | March 2009 |
| M02006 | M02 | Water | March 2009 |
| M02007 | M02 | Water | June 2009 |
| M02008 | M02 | Water | June 2009 |
| M02009 | M02 | Water | December 2009 |
| M02010 | M02 | Water | December 2009 |
| M02014 | M02 | Water | March 2010 |
| M02016 | M02 | Water | March 2010 |
| M02017 | M02 | Water | March 2010 |
| M02018 | M02 | Water | March 2010 |
| M02024 | M02 | Sediment | June 2010 |
| M02025 | M02 | Sediment | June 2010 |
| M02026 | M02 | Sediment | June 2010 |
| M02029 | M02 | Sediment | June 2010 |
| M02030 | M02 | Sediment | June 2010 |
| M02031 | M02 | Sediment | June 2010 |
| M02032 | M02 | Sediment | June 2010 |
| M02034 | M02 | Sediment | June 2010 |
| M02035 | M02 | Sediment | June 2010 |
| M02036 | M02 | Sediment | June 2010 |
| M02038 | M02 | Sediment | June 2010 |
| M02039 | M02 | Sediment | June 2010 |
| M02040 | M02 | Sediment | June 2010 |
| M02048 | M02 | Water | March 2011 |
| M02049 | M02 | Water | March 2011 |
| M02050 | M02 | Water | March 2011 |
| M02051 | M02 | Water | March 2011 |
| M03001 | M03 | Water | March 2009 |
| M03003 | M03 | Water | March 2009 |
| M03006 | M03 | Water | March 2009 |
| M03008 | M03 | Water | March 2009 |
| M03010 | M03 | Water | June 2009 |
| M03011 | M03 | Water | June 2009 |
| M03012 | M03 | Water | June 2009 |
| M03013 | M03 | Water | December 2009 |
| M03014 | M03 | Water | December 2009 |
| M03015 | M03 | Water | December 2009 |
| M03016 | M03 | Water | December 2009 |
| M03017 | M03 | Water | December 2009 |
| M03018 | M03 | Water | December 2009 |
| M03019 | M03 | Water | December 2009 |
| M03020 | M03 | Water | December 2009 |
| M03021 | M03 | Water | December 2009 |
| M03022 | M03 | Water | December 2009 |
| M03023 | M03 | Water | December 2009 |
| M03024 | M03 | Water | December 2009 |
| M03025 | M03 | Water | December 2009 |
| M03026 | M03 | Water | December 2009 |
| M03028 | M03 | Water | March 2011 |
| M03029 | M03 | Water | March 2011 |
| M03030 | M03 | Water | March 2011 |
| M03031 | M03 | Water | March 2011 |
| M03032 | M03 | Water | March 2011 |
| M03039 | M03 | Water | March 2011 |
| M03047 | M03 | Water | March 2011 |
| M03048 | M03 | Water | March 2011 |
| M04002 | M04 | Water | March 2009 |
| M04003 | M04 | Water | March 2009 |
| M04004 | M04 | Water | March 2009 |
| M04005 | M04 | Water | March 2009 |
| M04006 | M04 | Water | March 2009 |
| M04007 | M04 | Water | March 2009 |
| M04008 | M04 | Water | March 2009 |
| M04009 | M04 | Water | June 2009 |
| M04010 | M04 | Water | June 2009 |
| M04011 | M04 | Water | June 2009 |
| M04012 | M04 | Water | June 2009 |
| M04013 | M04 | Water | June 2009 |
| M04014 | M04 | Water | December 2009 |
| M04015 | M04 | Water | December 2009 |
| M04017 | M04 | Water | December 2009 |
| M04018 | M04 | Water | December 2009 |
| M04019 | M04 | Water | December 2009 |
| M04022 | M04 | Water | December 2009 |
| M04023 | M04 | Water | December 2009 |
| M04024 | M04 | Water | December 2009 |
| M04025 | M04 | Water | December 2009 |
| M04027 | M04 | Water | March 2011 |
| M04029 | M04 | Water | March 2012 |
| M04032 | M04 | Water | March 2013 |
| M05001 | M05 | Water | March 2009 |
| M05002 | M05 | Water | March 2009 |
| M05003 | M05 | Water | March 2009 |
| M05004 | M05 | Water | March 2009 |
| M05005 | M05 | Water | March 2009 |
| M05006 | M05 | Water | March 2009 |
| M05007 | M05 | Water | March 2009 |
| M05009 | M05 | Water | March 2009 |
| M05010 | M05 | Water | March 2009 |
| M05012 | M05 | Water | March 2009 |
| M05015 | M05 | Water | March 2009 |
| M05016 | M05 | Water | March 2009 |
| M05020 | M05 | Water | June 2009 |
| M05021 | M05 | Water | June 2009 |
| M05022 | M05 | Water | June 2009 |
| M05023 | M05 | Water | June 2009 |
| M05024 | M05 | Water | June 2009 |
| M05028 | M05 | Water | December 2009 |
| M05031 | M05 | Water | December 2009 |
| M05032 | M05 | Water | December 2009 |
| M05033 | M05 | Water | December 2009 |
| M05034 | M05 | Water | December 2009 |
| M05035 | M05 | Water | March 2011 |
| M05036 | M05 | Water | March 2011 |
| M05037 | M05 | Water | March 2011 |
| M05038 | M05 | Water | March 2011 |
| M05042 | M05 | Water | March 2011 |
| M06001 | M06 | Water | March 2009 |
| M06002 | M06 | Water | March 2009 |
| M06003 | M06 | Water | March 2009 |
| M06005 | M06 | Water | June 2009 |
| M06006 | M06 | Water | December 2009 |
| M06007 | M06 | Water | December 2009 |
| M06008 | M06 | Water | March 2011 |
| M07004 | M07 | Water | March 2009 |
| M07006 | M07 | Water | June 2009 |
| M07007 | M07 | Sediment | December 2009 |
| M07008 | M07 | Sediment | December 2009 |
| M07009 | M07 | Sediment | June 2010 |
| M07010 | M07 | Sediment | June 2010 |
| M07011 | M07 | Sediment | November 2010 |
| M07013 | M07 | Water | March 2011 |
| M07014 | M07 | Water | March 2011 |
| M08001 | M08 | Water | June 2009 |
| M08002 | M08 | Water | June 2009 |
| M08003 | M08 | Water | December 2009 |
| M08004 | M08 | Water | December 2009 |
| M08005 | M08 | Water | December 2009 |
| M08006 | M08 | Water | December 2009 |
| M08007 | M08 | Water | December 2009 |
| M08008 | M08 | Water | March 2010 |
| M08009 | M08 | Water | March 2010 |
| M08010 | M08 | Water | March 2010 |
| M08016 | M08 | Sediment | June 2010 |
| M08017 | M08 | Sediment | June 2010 |
| M08018 | M08 | Sediment | June 2010 |
| M08019 | M08 | Sediment | June 2010 |
| M08020 | M08 | Sediment | June 2010 |
| M08021 | M08 | Sediment | November 2010 |
| M08022 | M08 | Sediment | November 2010 |
| M08023 | M08 | Sediment | November 2010 |
| M08024 | M08 | Sediment | November 2010 |
| M08027 | M08 | Sediment | November 2010 |
| M08028 | M08 | Sediment | November 2010 |
| M09001 | M09 | Water | March 2009 |
| M09002 | M09 | Water | March 2009 |
| M09003 | M09 | Water | March 2009 |
| M09004 | M09 | Water | March 2009 |
| M09005 | M09 | Water | March 2009 |
| M09006 | M09 | Water | June 2009 |
| M09007 | M09 | Water | June 2009 |
| M09008 | M09 | Water | June 2009 |
| M09009 | M09 | Water | December 2009 |
| M09010 | M09 | Water | December 2009 |
| M09011 | M09 | Water | December 2009 |
| M09013 | M09 | Water | December 2009 |
| M09014 | M09 | Water | December 2009 |
| M09015 | M09 | Water | December 2009 |
| M09016 | M09 | Sediment | March 2010 |
| M09017 | M09 | Sediment | March 2010 |
| M09018 | M09 | Sediment | March 2010 |
| M09019 | M09 | Sediment | March 2010 |
| M09021 | M09 | Sediment | March 2010 |
| M09022 | M09 | Sediment | March 2010 |
| M09023 | M09 | Sediment | March 2010 |
| M09025 | M09 | Sediment | March 2010 |
| M09026 | M09 | Water | March 2011 |
| M10002 | M10 | Water | June 2009 |
| M10003 | M10 | Water | June 2009 |
| M10005 | M10 | Water | March 2011 |
| M10006 | M10 | Water | March 2011 |
| M11001 | M11 | Water | June 2009 |
| M11002 | M11 | Water | June 2009 |
| M11003 | M11 | Water | March 2011 |
| M11005 | M11 | Water | March 2011 |
| M12001 | M12 | Water | June 2009 |
| M12003 | M12 | Sediment | March 2010 |
| M12005 | M12 | Sediment | March 2010 |
| M12007 | M12 | Sediment | March 2010 |
| M12008 | M12 | Sediment | March 2010 |
| M12010 | M12 | Sediment | June 2010 |
| M12011 | M12 | Sediment | June 2010 |
| M12012 | M12 | Sediment | June 2010 |
| M12013 | M12 | Sediment | June 2010 |
| M12015 | M12 | Sediment | November 2010 |
| M12019 | M12 | Sediment | November 2010 |
| M12020 | M12 | Water | March 2011 |
| M12021 | M12 | Water | March 2011 |
| M12022 | M12 | Water | March 2011 |
| M13001 | M13 | Water | March 2009 |
| M13002 | M13 | Water | March 2009 |
| M13003 | M13 | Water | March 2009 |
| M13005 | M13 | Water | March 2009 |
| M13006 | M13 | Water | March 2009 |
| M13007 | M13 | Water | June 2009 |
| M13008 | M13 | Water | June 2009 |
| M13009 | M13 | Water | March 2011 |
| M13010 | M13 | Water | March 2011 |
| M14001 | M14 | Water | March 2009 |
| M14002 | M14 | Water | June 2009 |
| M14003 | M14 | Water | June 2009 |
| M14006 | M14 | Sediment | June 2009 |
| M14007 | M14 | Sediment | June 2009 |
| M14009 | M14 | Sediment | June 2009 |
| M14010 | M14 | Water | December 2009 |
| M14012 | M14 | Water | December 2009 |
| M14013 | M14 | Water | December 2009 |
| M14020 | M14 | Water | March 2011 |
| M15001 | M15 | Water | March 2009 |
| M15002 | M15 | Water | March 2009 |
| M15007 | M15 | Water | June 2009 |
| M15008 | M15 | Water | June 2009 |
| M15011 | M15 | Water | June 2009 |
| M15012 | M15 | Sediment | March 2010 |
| M15013 | M15 | Sediment | March 2010 |
| M15014 | M15 | Sediment | March 2010 |
| M15015 | M15 | Sediment | March 2010 |
| M15016 | M15 | Sediment | March 2010 |
| M15017 | M15 | Sediment | March 2010 |
| M15018 | M15 | Sediment | March 2010 |
| M15019 | M15 | Sediment | March 2010 |
| M15021 | M15 | Sediment | March 2010 |
| M15022 | M15 | Sediment | March 2010 |
| M15024 | M15 | Sediment | June 2010 |
| M15025 | M15 | Sediment | November 2010 |
| M15026 | M15 | Sediment | November 2010 |
| M15027 | M15 | Water | March 2011 |
| M15032 | M15 | Water | March 2011 |
| M15038 | M15 | Sediment | March 2011 |
| M16001 | M16 | Water | March 2009 |
| M16002 | M16 | Sediment | June 2009 |
| M16003 | M16 | Sediment | June 2009 |
| M16005 | M16 | Sediment | June 2009 |
| M16006 | M16 | Sediment | June 2009 |
| M16017 | M16 | Sediment | June 2009 |
| M16034 | M16 | Sediment | December 2009 |
| M16035 | M16 | Sediment | December 2009 |
| M16036 | M16 | Sediment | December 2009 |
| M16040 | M16 | Sediment | December 2009 |
| M16043 | M16 | Sediment | December 2009 |
| M16044 | M16 | Sediment | December 2009 |
| M16048 | M16 | Sediment | December 2009 |
| M16049 | M16 | Water | March 2011 |
| M18001 | M18 | Water | March 2009 |
| M18002 | M18 | Water | March 2009 |
| M18003 | M18 | Water | June 2009 |
| M18004 | M18 | Water | December 2009 |
| M18007 | M18 | Sediment | March 2010 |
| M18008 | M18 | Sediment | March 2010 |
| M18009 | M18 | Sediment | March 2010 |
| M18010 | M18 | Sediment | March 2010 |
| M18011 | M18 | Sediment | March 2010 |
| M18012 | M18 | Sediment | March 2010 |
| M18013 | M18 | Sediment | March 2010 |
| M18014 | M18 | Sediment | March 2010 |
| M18015 | M18 | Sediment | June 2010 |
| M18018 | M18 | Sediment | June 2010 |
| M18020 | M18 | Sediment | June 2010 |
| M18021 | M18 | Sediment | November 2010 |
| M18022 | M18 | Sediment | March 2011 |
| M18023 | M18 | Sediment | March 2011 |
| M18026 | M18 | Water | March 2011 |
| M19001 | M19 | Water | March 2009 |
| M19002 | M19 | Water | March 2009 |
| M19004 | M19 | Water | March 2009 |
| M19005 | M19 | Water | June 2009 |
| M19006 | M19 | Sediment | June 2009 |
| M19007 | M19 | Sediment | June 2009 |
| M19008 | M19 | Sediment | June 2009 |
| M19009 | M19 | Sediment | June 2009 |
| M19011 | M19 | Sediment | June 2009 |
| M19014 | M19 | Sediment | June 2009 |
| M19015 | M19 | Sediment | June 2009 |
| M19016 | M19 | Sediment | June 2009 |
| M19017 | M19 | Sediment | June 2009 |
| M19018 | M19 | Sediment | June 2009 |
| M19019 | M19 | Sediment | June 2009 |
| M19021 | M19 | Sediment | June 2009 |
| M19022 | M19 | Sediment | June 2009 |
| M19024 | M19 | Sediment | June 2009 |
| M19027 | M19 | Sediment | June 2009 |
| M19028 | M19 | Water | December 2009 |
| M19029 | M19 | Water | December 2009 |
| M19039 | M19 | Water | December 2009 |
| M19041 | M19 | Water | December 2009 |
| M19043 | M19 | Water | December 2009 |
| M19044 | M19 | Water | December 2009 |
| M19045 | M19 | Water | December 2009 |
| M19047 | M19 | Water | December 2009 |
| M19050 | M19 | Sediment | December 2009 |
| M19051 | M19 | Sediment | December 2009 |
| M19053 | M19 | Sediment | December 2009 |
| M19054 | M19 | Sediment | December 2009 |
| M19056 | M19 | Sediment | December 2009 |
| M19057 | M19 | Sediment | December 2009 |
| M19064 | M19 | Sediment | December 2009 |
| M19065 | M19 | Sediment | December 2009 |
| M19066 | M19 | Sediment | December 2009 |
| M19067 | M19 | Sediment | December 2009 |
| M19068 | M19 | Sediment | December 2009 |
| M19069 | M19 | Sediment | December 2009 |
| M19070 | M19 | Sediment | December 2009 |
| M19072 | M19 | Sediment | December 2009 |
| M19074 | M19 | Sediment | December 2009 |
| M19076 | M19 | Sediment | December 2009 |
| M19078 | M19 | Sediment | December 2009 |
| M19080 | M19 | Sediment | December 2009 |
| M19082 | M19 | Water | March 2010 |
| M19083 | M19 | Water | March 2010 |
| M19090 | M19 | Water | March 2011 |
| M21001 | M21 | Water | March 2009 |
| M21002 | M21 | Water | June 2009 |
| M21005 | M21 | Water | June 2009 |
| M21008 | M21 | Water | June 2009 |
| M21023 | M21 | Water | December 2009 |
| M21024 | M21 | Water | December 2009 |
| M21027 | M21 | Water | December 2009 |
| M21028 | M21 | Water | December 2009 |
| M21032 | M21 | Sediment | March 2010 |
| M21034 | M21 | Sediment | March 2010 |
| M21035 | M21 | Sediment | March 2010 |
| M21036 | M21 | Sediment | March 2010 |
| M21039 | M21 | Water | March 2011 |
| M21040 | M21 | Water | March 2011 |
