# Supplementary material for: Metal tolerance of Río Tinto fungi
Source: Front Fungal Biol. 2024 Oct 16;5:1446674. doi: 10.3389/ffunb.2024.1446674 (PMC11521807; doi:10.3389/ffunb.2024.1446674)
Supplement: Supplementary file 7 [file Table4.docx]

**Table S4. Fungal classes presence/absence and information data (sampling station, year and season) for each sample.**

| **Sample** | ***Agaricomycetes*** | ***Ascomycota*_unknown** | ***Dothideomycetes*** | ***Eurotiomycetes*** | ***Leotiomycetes*** | ***Mortierellomycetes*** | ***Mucoromycetes*** | ***Sordariomycetes*** | ***Umbelopsidomycetes*** | **Station** | **Date** |
| --- | --- | --- | --- | --- | --- | --- | --- | --- | --- | --- | --- |
| M01.1 | 0 | 0 | 1 | 0 | 0 | 0 | 0 | 1 | 0 | M01 | March 2009 |
| M01.2 | 0 | 0 | 1 | 0 | 0 | 0 | 0 | 0 | 0 | M01 | June 2009 |
| M01.3 | 0 | 0 | 1 | 1 | 0 | 0 | 0 | 0 | 0 | M01 | December 2009 |
| M01.5 | 0 | 0 | 1 | 0 | 0 | 0 | 0 | 1 | 0 | M01 | June 2010 |
| M01.6 | 0 | 0 | 1 | 1 | 0 | 0 | 0 | 1 | 0 | M01 | November 2010 |
| M01.7 | 0 | 0 | 1 | 1 | 0 | 0 | 1 | 1 | 0 | M01 | March 2011 |
| M02.1 | 0 | 0 | 0 | 1 | 0 | 1 | 0 | 0 | 0 | M02 | March 2009 |
| M02.2 | 0 | 1 | 1 | 0 | 0 | 0 | 0 | 0 | 0 | M02 | June 2009 |
| M02.3 | 0 | 0 | 1 | 0 | 0 | 0 | 0 | 0 | 0 | M02 | December 2009 |
| M02.4 | 0 | 0 | 0 | 1 | 0 | 0 | 0 | 1 | 0 | M02 | March 2010 |
| M02.5 | 0 | 0 | 0 | 1 | 0 | 0 | 0 | 1 | 0 | M02 | June 2010 |
| M02.7 | 0 | 0 | 1 | 0 | 0 | 0 | 0 | 1 | 0 | M02 | March 2011 |
| M03.1 | 0 | 0 | 0 | 1 | 1 | 0 | 0 | 0 | 0 | M03 | March 2009 |
| M03.2 | 0 | 0 | 1 | 1 | 0 | 0 | 0 | 0 | 0 | M03 | June 2009 |
| M03.3 | 0 | 0 | 1 | 1 | 0 | 0 | 0 | 1 | 0 | M03 | December 2009 |
| M03.7 | 0 | 0 | 0 | 1 | 0 | 0 | 0 | 1 | 0 | M03 | March 2011 |
| M04.1 | 0 | 0 | 1 | 1 | 0 | 0 | 0 | 0 | 1 | M04 | March 2009 |
| M04.2 | 0 | 0 | 0 | 1 | 0 | 0 | 0 | 1 | 0 | M04 | June 2009 |
| M04.3 | 0 | 0 | 0 | 1 | 0 | 0 | 0 | 1 | 0 | M04 | December 2009 |
| M04.7 | 0 | 0 | 0 | 0 | 0 | 0 | 1 | 1 | 0 | M04 | March 2011 |
| M05.1 | 0 | 0 | 1 | 1 | 0 | 0 | 0 | 1 | 0 | M05 | March 2009 |
| M05.2 | 0 | 0 | 1 | 1 | 1 | 0 | 0 | 0 | 0 | M05 | June 2009 |
| M05.3 | 0 | 0 | 1 | 1 | 0 | 0 | 0 | 0 | 0 | M05 | December 2009 |
| M05.7 | 0 | 0 | 0 | 0 | 0 | 0 | 0 | 1 | 0 | M05 | March 2011 |
| M06.1 | 0 | 0 | 1 | 0 | 0 | 0 | 0 | 0 | 0 | M06 | March 2009 |
| M06.2 | 0 | 0 | 1 | 0 | 0 | 0 | 0 | 0 | 0 | M06 | June 2009 |
| M06.3 | 0 | 0 | 1 | 0 | 0 | 0 | 0 | 0 | 0 | M06 | December 2009 |
| M06.7 | 0 | 0 | 1 | 0 | 0 | 0 | 0 | 0 | 0 | M06 | March 2011 |
| M07.1 | 0 | 0 | 1 | 0 | 0 | 0 | 0 | 0 | 0 | M07 | March 2009 |
| M07.2 | 0 | 0 | 1 | 0 | 0 | 0 | 0 | 0 | 0 | M07 | June 2009 |
| M07.3 | 0 | 0 | 1 | 0 | 0 | 0 | 0 | 1 | 0 | M07 | December 2009 |
| M07.5 | 0 | 0 | 1 | 0 | 0 | 0 | 0 | 0 | 0 | M07 | June 2010 |
| M07.6 | 0 | 0 | 0 | 1 | 0 | 0 | 0 | 0 | 0 | M07 | November 2010 |
| M07.7 | 0 | 0 | 1 | 0 | 0 | 0 | 0 | 1 | 0 | M07 | March 2011 |
| M08.2 | 0 | 0 | 0 | 1 | 0 | 0 | 0 | 1 | 0 | M08 | June 2009 |
| M08.3 | 0 | 0 | 1 | 0 | 0 | 0 | 0 | 0 | 0 | M08 | December 2009 |
| M08.4 | 0 | 0 | 0 | 1 | 0 | 0 | 0 | 1 | 0 | M08 | March 2010 |
| M08.5 | 0 | 0 | 1 | 1 | 0 | 0 | 0 | 1 | 0 | M08 | June 2010 |
| M08.6 | 0 | 0 | 1 | 1 | 0 | 0 | 0 | 0 | 0 | M08 | November 2010 |
| M09.1 | 0 | 0 | 1 | 1 | 0 | 0 | 0 | 0 | 0 | M09 | March 2009 |
| M09.2 | 0 | 0 | 1 | 1 | 0 | 0 | 0 | 0 | 0 | M09 | June 2009 |
| M09.3 | 0 | 0 | 1 | 0 | 0 | 0 | 0 | 0 | 0 | M09 | December 2009 |
| M09.4 | 0 | 0 | 1 | 1 | 1 | 0 | 0 | 0 | 0 | M09 | March 2010 |
| M09.7 | 0 | 0 | 1 | 0 | 0 | 0 | 0 | 0 | 0 | M09 | March 2011 |
| M10.2 | 0 | 0 | 0 | 0 | 1 | 0 | 0 | 0 | 0 | M10 | June 2009 |
| M10.5 | 0 | 0 | 0 | 1 | 0 | 0 | 0 | 0 | 0 | M10 | June 2010 |
| M10.7 | 0 | 0 | 0 | 1 | 0 | 0 | 0 | 0 | 0 | M10 | March 2011 |
| M11.2 | 0 | 0 | 1 | 0 | 1 | 0 | 0 | 0 | 0 | M11 | June 2009 |
| M11.7 | 0 | 0 | 1 | 1 | 0 | 0 | 0 | 0 | 0 | M11 | March 2011 |
| M12.2 | 0 | 0 | 1 | 0 | 0 | 0 | 0 | 0 | 0 | M12 | June 2009 |
| M12.4 | 0 | 0 | 0 | 1 | 0 | 0 | 0 | 0 | 0 | M12 | March 2010 |
| M12.5 | 0 | 0 | 0 | 1 | 0 | 0 | 0 | 0 | 0 | M12 | June 2010 |
| M12.6 | 0 | 0 | 0 | 1 | 0 | 0 | 0 | 1 | 0 | M12 | November 2010 |
| M12.7 | 0 | 0 | 1 | 1 | 0 | 0 | 0 | 0 | 0 | M12 | March 2011 |
| M13.1 | 0 | 1 | 0 | 1 | 0 | 0 | 0 | 1 | 0 | M13 | March 2009 |
| M13.2 | 0 | 0 | 1 | 1 | 0 | 0 | 0 | 0 | 0 | M13 | June 2009 |
| M13.7 | 0 | 0 | 0 | 0 | 0 | 0 | 0 | 1 | 0 | M13 | March 2011 |
| M14.1 | 0 | 0 | 0 | 1 | 0 | 0 | 0 | 0 | 0 | M14 | March 2009 |
| M14.2 | 0 | 0 | 1 | 1 | 0 | 0 | 0 | 0 | 0 | M14 | June 2009 |
| M14.3 | 0 | 0 | 1 | 1 | 0 | 0 | 0 | 0 | 0 | M14 | December 2009 |
| M14.4 | 0 | 0 | 0 | 1 | 0 | 0 | 0 | 0 | 0 | M14 | March 2010 |
| M14.7 | 0 | 0 | 0 | 1 | 0 | 0 | 0 | 0 | 0 | M14 | March 2011 |
| M15.1 | 0 | 0 | 0 | 0 | 0 | 0 | 0 | 1 | 0 | M15 | March 2009 |
| M15.2 | 0 | 0 | 1 | 1 | 1 | 0 | 0 | 0 | 0 | M15 | June 2009 |
| M15.4 | 1 | 0 | 1 | 1 | 0 | 0 | 0 | 1 | 0 | M15 | March 2010 |
| M15.5 | 0 | 0 | 0 | 1 | 0 | 0 | 0 | 0 | 0 | M15 | June 2010 |
| M15.6 | 0 | 0 | 0 | 1 | 0 | 0 | 0 | 0 | 0 | M15 | November 2010 |
| M15.7 | 0 | 0 | 1 | 0 | 0 | 0 | 0 | 1 | 0 | M15 | March 2011 |
| M16.1 | 0 | 0 | 0 | 1 | 0 | 0 | 0 | 0 | 0 | M16 | March 2009 |
| M16.2 | 0 | 0 | 0 | 1 | 0 | 0 | 0 | 0 | 0 | M16 | June 2009 |
| M16.3 | 0 | 0 | 1 | 1 | 0 | 0 | 0 | 0 | 0 | M16 | December 2009 |
| M16.7 | 0 | 0 | 0 | 0 | 0 | 0 | 0 | 1 | 0 | M16 | March 2011 |
| M18.1 | 0 | 0 | 1 | 1 | 0 | 0 | 0 | 0 | 0 | M18 | March 2009 |
| M18.2 | 0 | 0 | 0 | 1 | 0 | 0 | 0 | 0 | 0 | M18 | June 2009 |
| M18.3 | 0 | 0 | 0 | 1 | 0 | 0 | 0 | 0 | 0 | M18 | December 2009 |
| M18.4 | 0 | 0 | 0 | 1 | 0 | 0 | 0 | 1 | 0 | M18 | March 2010 |
| M18.5 | 0 | 0 | 1 | 1 | 0 | 0 | 0 | 0 | 0 | M18 | June 2010 |
| M18.7 | 0 | 0 | 0 | 0 | 0 | 0 | 0 | 1 | 0 | M18 | March 2011 |
| M19.1 | 1 | 1 | 1 | 0 | 0 | 0 | 0 | 0 | 0 | M19 | March 2009 |
| M19.2 | 1 | 0 | 0 | 1 | 0 | 0 | 0 | 1 | 0 | M19 | June 2009 |
| M19.3 | 1 | 0 | 1 | 1 | 0 | 0 | 0 | 1 | 0 | M19 | December 2009 |
| M19.4 | 0 | 0 | 0 | 1 | 0 | 0 | 0 | 1 | 0 | M19 | March 2010 |
| M19.7 | 0 | 0 | 0 | 1 | 0 | 0 | 0 | 0 | 0 | M19 | March 2011 |
| M21.1 | 0 | 0 | 1 | 0 | 0 | 0 | 0 | 0 | 0 | M21 | March 2009 |
| M21.2 | 0 | 0 | 0 | 1 | 0 | 0 | 0 | 0 | 0 | M21 | June 2009 |
| M21.3 | 0 | 0 | 1 | 1 | 0 | 0 | 0 | 0 | 0 | M21 | December 2009 |
| M21.4 | 0 | 0 | 0 | 1 | 0 | 0 | 0 | 1 | 0 | M21 | March 2010 |
| M21.7 | 0 | 0 | 1 | 1 | 0 | 0 | 0 | 0 | 0 | M21 | March 2011 |
