# Supplementary material for: Metal tolerance of Río Tinto fungi
Source: Front Fungal Biol. 2024 Oct 16;5:1446674. doi: 10.3389/ffunb.2024.1446674 (PMC11521807; doi:10.3389/ffunb.2024.1446674)
Supplement: Supplementary file 8 [file Table5.docx]

**Table S5. Shannon and Chao 1 diversity indexes.** The alpha diversity indexes have been calculated as described in the Materials and Methods section.

| **Sample** | **Shannon index** | **Chao1 index** | **N Obs** |
| --- | --- | --- | --- |
| M01 | 0,77 | 30,5 | 29 |
| M02 | 0,92 | 66 | 30 |
| M03 | 0,75 | 29,5 | 29 |
| M04 | 0,62 | 48,5 | 24 |
| M05 | 0,80 | 59 | 27 |
| M06 | 0,35 | 8 | 7 |
| M07 | 0,47 | 11 | 8 |
| M08 | 0,67 | 23 | 21 |
| M09 | 0,55 | 24,5 | 24 |
| M10 | 0,28 | 3,5 | 3 |
| M11 | 0,45 | 6 | 4 |
| M12 | 0,61 | 17,25 | 15 |
| M13 | 0,62 | 13,5 | 9 |
| M14 | 0,53 | 10,25 | 10 |
| M15 | 1,03 | 27 | 21 |
| M16 | 0,39 | 16 | 14 |
| M18 | 0,75 | 23,5 | 19 |
| M19 | 0,73 | 66 | 48 |
| M21 | 0,67 | 15 | 15 |
